# Bio-barrier-adaptable biomimetic nanomedicines combined with ultrasound for enhanced cancer therapy

**DOI:** 10.1038/s41392-025-02217-8

**Published:** 2025-04-25

**Authors:** Juan Guo, Xueting Pan, Qingyuan Wu, Ping Li, Chaohui Wang, Shuang Liu, Haoyuan Zhang, Zezhong Huang, Xiaozhou Mou, Huiyu Liu, Jiajia Xue

**Affiliations:** 1 Beijing Advanced Innovation Center for Soft Matter Science and Engineering, State Key Laboratory of Organic-Inorganic Composites, Beijing Laboratory of Biomedical Materials, Bionanomaterials & Translational Engineering Laboratory, Beijing Key Laboratory of Bioprocess, Beijing University of Chemical Technology, Beijing, China; 2 Clinical Research Institute, Zhejiang provincial People’s Hospital (Affiliated People’s Hospital), Hangzhou Medical College, Hangzhou, Zhejiang Province China

**Keywords:** Cancer therapy, Drug delivery

## Abstract

Addressing the critical biological barriers of targeted accumulation and deep tumor penetration remains essential for the clinical translation of nanomedicines. However, existing nanomedicines often face challenges during in vivo transportation, including immune clearance, tumor microenvironmental barriers, and limited vascular permeability, which collectively reduce drug delivery efficiency and compromise therapeutic efficacy. Here, we present a bio-barrier-adaptable biomimetic nanoplatform, MSF@CCM, which integrates a mesoporous silica-loaded iron oxyhydroxide (MSF) core camouflaged with a homologous membrane. This design conferred dual functionality: (1) enhanced tumor accumulation and immune evasion by exploiting homologous cell-cell interactions and mimicking “self” markers, thereby effectively bypassing macrophage clearance and surpassing the limitations of traditional targeted drug delivery; and (2) amplified ultrasound (US)-mediated intratumoral penetration. The MSF core, with its unique porous structure and rough surface, significantly enhanced US cavitation effects, transiently disrupting tumor vasculature and facilitating deep penetration of nanomedicines. Upon US triggering, MSF@CCM effectively disrupted intracellular redox homeostasis, potently inducing ferroptosis via lipid peroxidation accumulation, mitochondrial morphological changes, and decreased key protein expression. This combined therapeutic strategy achieved a remarkable 96.5% tumor growth inhibition in vivo while maintaining favorable biocompatibility. Our findings establish a novel paradigm for overcoming multidimensional bio-barriers through biohybrid engineering and physical energy synergy, offering a promising modality for enhanced cancer therapy.

## Introduction

Navigating the complex biological barriers of solid tumors presents a formidable challenge for effective nanomedicine delivery.^1^ Key obstacles encompassing the vasculature, dense extracellular matrix (ECM), elevated interstitial fluid pressure, and hypoxic tumor microenvironment, which pose significant threats to the therapeutic efficacy of nanomedicine.^2^ In detail, abnormal and disorganized tumor vasculature typically results in uneven blood flow, and formation of hypoxic and acidic tumor microenvironment, which in turn leads to tumor progression, inefficient drug accumulation, and compromised therapeutic efficacy. Meanwhile, the high interstitial fluid pressure within the tumor also acts as a countervailing force that prevents nanomedicines from effectively penetrating into the deeper tumor regions.^3^ Although the enhanced permeation and retention (EPR) effect offers a theoretical basis for passive tumor accumulation, its efficacy is limited by tumor heterogeneity and the high variability of tumor vasculature.^4,5^ Furthermore, conventional targeted delivery strategies are often hindered by the dynamic and heterogeneous expression of target moieties on tumor cells, leading to suboptimal targeting efficiency and, in some cases, induced resistance due to clonal selection pressure.^6,7^ Consequently, innovative approaches are crucial for designing advanced nanomedicines for adapting the complex tumor microenvironment, enhanced tumor penetration, and therapeutic efficacy.

Biomimetic drug delivery systems represent a promising strategy to address these challenges by enhancing tumor targeting.^8,9^ Cell membrane-camouflaged nanoparticles combine the drug-carrying capacity of nanoparticles with the inherent biological functionality of cell membranes, facilitating improved circulation time, homotypic targeting, and immune evasion, thus enhancing tumor accumulation.^10,11^ Adhesion molecules such as N-cadherin, E-cadherin, and EpCAM, which are abundantly present on cell membrane, facilitate homotypic interactions and promote selective tumor accumulation.^12–14^ Meanwhile, immune evasion markers (e.g., CD47), enable nanoparticles to mimic “self” signals, effectively reducing clearance by the reticuloendothelial system (RES) and extending systemic circulation time, ultimately enhancing drug accumulation at the tumor site.^15^ Moreover, biomimetic platforms can be further engineered through genetic modifications or chemical functionalization to improve adaptability to tumor heterogeneity and enable stimulus-responsive drug release, offering a versatile strategy for precision nanomedicine. With the continuous development of bio-nanotechnology, the integration of multiple functional biomimetic coatings (e.g., hybridization of tumor cell membranes and immune cell membranes) has been widely developed.^16^ The application of hybrid membrane coatings can further enhance the tumor-specific accumulation of nanomedicines and remodel the tumor immunosuppressive microenvironment, providing a new approach to improve the therapeutic efficiency and promote the tumor immune response.

Ultrasound (US)-mediated drug delivery is a rapidly evolving field that can further augment the penetration of nanomedicines cross biological barriers by overcoming vascular, ECM, and interstitial fluid pressure constraints.^17^ The core mechanism of this approach relies on the cavitation effect, where US exposure induces rapid oscillation and collapse of microbubbles or nanobubbles, generating high-energy shock waves and transiently creating a high-temperature, high-pressure microenvironment.^18^ This process temporarily enhances vascular permeability, disrupts the tumor stroma, reduces ECM-mediated physical resistance, promotes cellular uptake, and facilitates deeper drug penetration into tumors.^19–21^ Beyond mediating drug delivery, US could activate sonocatalytic nanomedicines to generate toxic reactive oxygen species (ROS), which directly induce DNA breaks, lipid peroxidation, and mitochondrial damage, ultimately leading to cell death.^22^ However, conventional sonocatalytic nanomedicines, often relying on single ROS generation mechanisms, face limitations in overcoming the intra-tumor barrier.^17^ Recent advancements in multifunctional sonocatalytic nanomaterials, such as those based on mesoporous silica nanoparticles (MSN), leverage porous structures and surface modifications to amplify cavitation effects, enhance ROS generation, and enhance tumor penetration.^23,24^ Furthermore, doping with metal ions or incorporating metal oxides can further enhance US responsiveness, promoting Fenton or Fenton-like reactions to induce potent oxidative stress and programmed cell death, such as ferroptosis.^25^ Integrating biomimetic strategies with US technology holds significant potential for improving drug delivery precision, achieving deeper tumor penetration, and ultimately enhancing therapeutic outcomes.

**Fig. 1:**
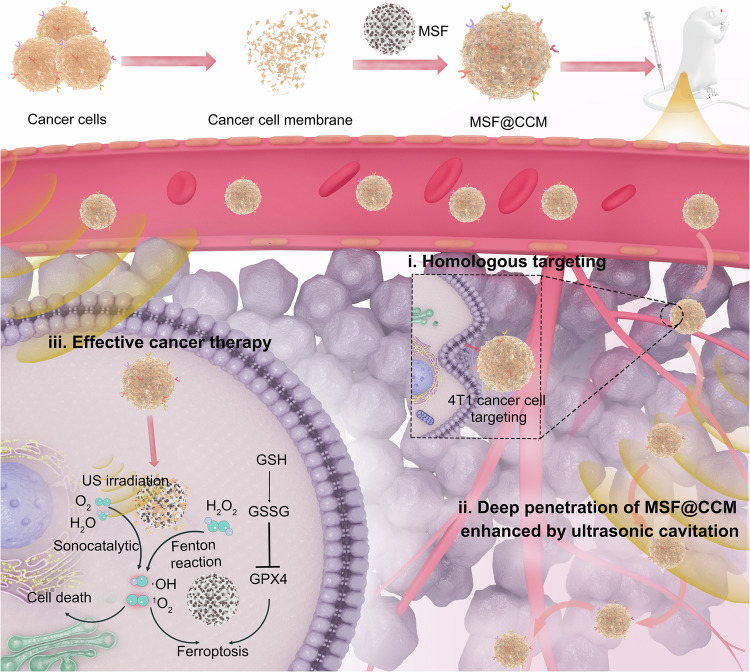
Schematic illustration of MSF@CCM synthesis, active targeting and US-mediated deep penetration for cancer therapy

Herein, we report a bio-barrier-adaptable nanomedicine platform comprising cancer cell membrane (CCM)-camouflaged mesoporous silica-FeOOH (MSF@CCM), designed for targeted tumor accumulation and enhanced therapeutic efficacy under US stimulation (Fig. 1). MSF@CCM combines the biomimetic properties of CCM for immune evasion by mimicking “self” markers (e.g., CD47) and tumor targeting through homotypic cell recognition, achieving 2.0-fold higher tumor retention than non-camouflaged nanomedicine. The MSF core leverages its porous structure and surface roughness to amplify US-mediated cavitation effects, generating localized mechanical forces that transiently disrupt tumor vascular endothelial gaps and ECM barriers, thereby promoting deep tumor penetration. The porous structure and surface properties of MSF enhance US-induced cavitation, leading to significantly higher ROS generation than FeOOH nanodots alone. Importantly, MSF@CCM initiates Fenton reactions to boost ROS generation, under US exposure, potentiates intracellular oxidative stress, triggering ferroptosis via lipid peroxidation (LPO) accumulation, mitochondrial morphological changes, downregulation of glutathione peroxidase 4 (GPX4) and long-chain acyl-CoA synthetase 4 (ACSL-4). In vivo studies demonstrate that US-activated MSF@CCM achieves 96.5% tumor suppression with negligible systemic toxicity. This platform exemplifies a rational integration of biomimetic camouflage and physical energy-responsive engineering, highlighting its potential as a bio-barrier-adaptable nanomedicine for improved cancer therapy.

## Results

### Preparation and characterization of MSF and MSF@CCM

**Fig. 2:**
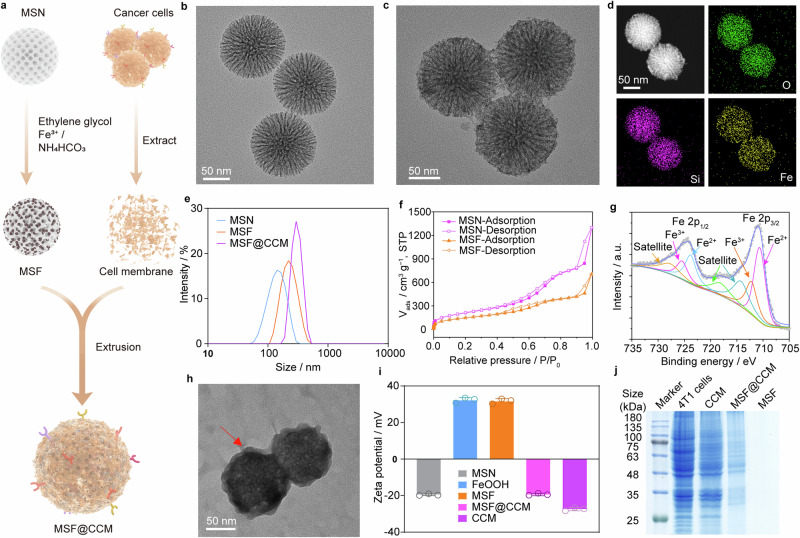
Schematic and characterization of MSF and MSF@CCM. **a** Preparation of MSF and MSF@CCM. TEM images of (**b**) MSN and (**c**) MSF. **d** Element mapping for O, Si and Fe of MSF. **e** The size distribution of MSN, MSF and MSF@CCM. **f** Nitrogen adsorption/desorption isotherms of MSN and MSF. **g** Core-level XPS spectrum of Fe 2p. **h** TEM image of MSF@CCM (the arrow indicates the cell membrane). **i** Zeta potentials of MSN, FeOOH nanodots, MSF, MSF@CCM, and CCM. Data are presented as mean ± SD (*n* = 3). **j** SDS-PAGE protein analysis of 4T1 cells, CCM, MSF@CCM and MSF

Uniform MSF was synthesized by improving the reported method using homogeneous chemical reactions.^26^ In detail, the MSN was simply introduced into the reaction system and used as a template to form amorphous MSF (Fig. 2a). From the transmission electron microscopy (TEM) images (Fig. 2b, c), the prepared MSN and MSF both had regular spherical morphologies, and the surface of the MSF appeared rougher than that of MSN, which could facilitate the stabilization of cavitation bubbles.^27^ The uniformly spherical morphology of MSF can also be observed from the scanning electron microscopy (SEM) images (Supplementary Fig. 1). The TEM and the particle size of FeOOH nanodots are shown in Supplementary Fig. 2. Meanwhile, the high-resolution transmission electron microscopy (HR-TEM) showed visible FeOOH nanodots, indicating the successful synthesis of the MSF composite (Supplementary Fig. 3). Furthermore, the elemental mapping showed that MSF contained uniformly distributed Fe, Si and O elements (Fig. 2d). The energy dispersive X-ray spectroscopy (EDS) data also confirmed that the main components were Fe, Si, and O (Supplementary Fig. 4). The content of Fe in MSF was about 33.21% determined by inductively coupled plasma mass spectrometry (ICP-MS). Dynamic light scattering (DLS) demonstrated that the hydrodynamic diameter of MSF was approximately 130 nm (Fig. 2e), which was beneficial to accumulate at the tumor sites by the EPR effect. Subsequently, the surface area and pore size distributions of the MSF were investigated. The pore size of pristine MSN was 2–12 nm while that of the MSF was 3–9 nm, indicating that FeOOH nanodots may be distributed in the pore channels in MSN (Supplementary Fig. 5). The Brunauer–Emmett–Teller (BET) specific surface area of MSN was 716.705 m^2^ g^−1^ and the pore volume was 1.953 m^3^ g^−1^, while that of MSF was 369.401 m^2^ g^−1^, and 0.978 m^3^ g^−1^, respectively (Fig. 2f). These results indicated that the MSF still retained a relatively large specific surface area and porous structure, which is potentially conducive to the cavitation effect and thus enhances ROS generation.^28^ The powder X-ray diffraction (XRD) pattern showed no diffraction peaks of the pristine FeOOH nanodots and MSF, revealing their amorphous structure (Supplementary Fig. 6). Amorphous materials have metastable electrons that can effectively promote the interfacial charge transfer process.^29^ Fourier-transform infrared spectroscopy (FTIR) pattern showed the distinctive peaks of Fe–OH bending vibrations and Fe–O stretching vibrations in FeOOH between 900 cm^−1^ and 500 cm^−1^, indicating the presence of FeOOH (Supplementary Fig. 7).^30,31^ In addition, the chemical state of the various bonding elements on the surface of MSF was further measured by X-ray photoelectron spectroscopy (XPS). The XPS full-survey-scan spectrum was shown in Supplementary Fig. 8a, indicating the coexistence of Fe, Si and O elements in the MSF. As shown in Fig. 2g, the core-level XPS spectrum of Fe 2p shows two major peaks located at 711.5 eV for Fe 2p_3/2_ and 724.5 eV for Fe 2p_1/2_. The Fe 2p_3/2_ spectrum can be divided into two major peaks at 710.7 eV and 712.4 eV for Fe^2+^ and Fe^3+^, respectively.^26,32^ There are two shake-up satellite peaks at 719.5 eV and 733.2 eV, indicating the presence of Fe^3+^.^33,34^ Deconvolution O 1 s core-level spectrum indicates the presence of three chemically distinct species, which peak at 531.6 eV, 529.9 eV and 532.7 eV for Fe–OH, Fe–O and H–O–H,^35,36^ respectively (Supplementary Fig. 8b). These results confirmed that amorphous FeOOH nanodots and MSF composites were successfully synthesized *via* a simple chemical reaction.

To achieve target accumulation and immune evasion, the CCM was used to camouflage MSF. In brief, the CCM was obtained by lysis and fractionated centrifugation, mixed with MSF, and then extruded through a series of polycarbonate membranes with pore sizes of 400 nm and 200 nm to obtain MSF@CCM (Fig. 2a). TEM image shows that MSF@CCM had uniformly spherical and irregular surface (Fig. 2h). The thickness of the CCM was about ~3 nm. Compared to MSF, the hydrodynamic diameter of MSF@CCM was slightly increased (Fig. 2e). Meanwhile, the surface zeta potential was changed from 32.9 mV to −18.9 mV because of the CCM coating (Fig. 2i). The colloidal stabilities of MSF and MSF@CCM under different conditions were observed. The results showed that the particle size of MSF@CCM in complete DMEM or PBS (pH 7.4) containing 0%, 10%, or 50% serum was not significantly changed within one week, while the particle size of MSF in PBS became progressively larger with time (Supplementary Fig. 9), suggesting that the camouflage of the CCM enhanced the colloidal stability, which was favorable for in vivo recycling and targeting accumulation. Furthermore, the sodium dodecyl sulfate-polyacrylamide gel electrophoresis (SDS-PAGE) and Western blotting were used to examine the membrane protein. The SDS-PAGE assay showed that the protein composition of MSF@CCM resembled that of CCM (Fig. 2j). The membrane proteins such as CD44 and CD47 were examined by Western blotting since they play a crucial role in cancer metastasis. CD44 is a critical adhesion molecule on the surface of 4T1 cells and plays a critical role in the adhesion of metastatic cells to distant sites.^37^ CD47, a self-marker molecule of 4T1 cells, which is closely associated with tumor aggressiveness through inhibiting phagocytosis of cancer cells by macrophages.^38^ As shown in Supplementary Fig. 10, 4T1 cell lysates, cell membranes and MSF@CCM, prepared by the same way as MSF@CCM, showed that both CD44 and CD47 marker proteins were retained. These results indicated that the proteins on CCM were successfully transferred to MSF@CCM. To further validate that MSF@CCM was successfully prepared, MSF and CCM were labeled with fluorescein isothiocyanate (FITC) and 1,1’-Dioctadecyl-3,3,3’,3’-Tetramethylindodicarbocyanine,4-Chlorobenzenesulfonate Salt (DiD), respectively. Confocal laser scanning microscopy (CLSM) images showed that the green fluorescence derived from MSF overlapped well with the red fluorescence derived from CCM (Supplementary Fig. 11), indicating the successful formation of the MSF@CCM. Collectively, these results suggested that the MSF cores were successfully wrapped by CCM.

### Homologous targeting and immune escape of MSF@CCM in vitro

**Fig. 3:**
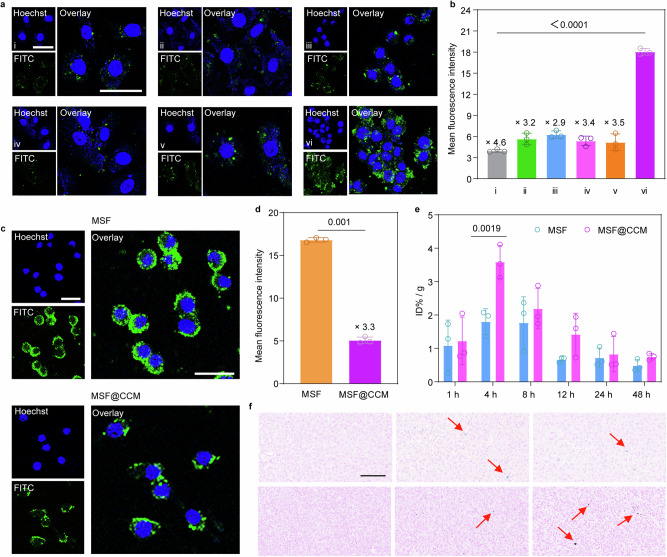
The homologous targeting capabilities and accumulation of MSF and MSF@CCM in vitro and in vivo. **a** CLSM images and (**b**) corresponding mean green fluorescence intensity of MSF@CCM incubated with (i) HUVEC, (ii) NIH-3T3, (iii) SCC, (iv) A549, (v) MDA-MB-231 and (vi) 4T1 cells. Scale bar = 25 μm. **c** CLSM images and (**d**) corresponding mean green fluorescence intensity of MSF and MSF@CCM incubated with RAW 264.7. Scale bar = 50 μm. **e** Tumor accumulation (based on Fe) of MSF and MSF@CCM in the 4T1 tumor-bearing mice after i.v. injection at different times. **f** Prussian blue staining of tumor after i.v. injection 4 h of MSF and MSF@CCM. Scale bar = 100 μm. Data are presented as mean ± SD (*n* = 3). One-way ANOVA with Tukey's multiple comparison was used to calculate statistical differences in (**b**, **d** and **e**)

Surface adhesion molecules expressed by cancer cells (*e.g*., EpCAM, CD44, and E-cadherin) play a crucial role in the homologous targeting process.^14^ To verify the homologous targeting abilities of MSF@CCM, the uptake of MSF@CCM was evaluated by normal cells and cancer cells, including human umbilical vein endothelial cells (HUVECs), and mouse embryonic cells (NIH-3T3), squamous cell carcinoma (SCC), human lung cancer cells (A549), human breast cancer cells (MDA-MB-231) and 4T1 cells, respectively (Fig. 3a). The FITC-labeled MSF (FITC-MSF) and FITC-labeled MSF@CCM (FITC-MSF@CCM) were further used. The fluorescence quantification results revealed that the uptake efficiency of the 4T1 cells group was much greater than that of other cell lines (Fig. 3b). Encouraged by these results, MSF@A549 and MSF@4T1 were incubated with 4T1 and A549, respectively, further demonstrating the highly specific targeting ability of MSF@CCM on homologous cancer cells (Supplementary Fig. 12). Furthermore, the tumor-associated antigens (e.g., CD47) on the CCM could prevent the uptake of cancer cells by macrophages through interaction with signal regulatory protein-α expressed on macrophages.^15^ Given this, the RAW 264.7 mouse macrophage was incubated with MSF and MSF@CCM to assess the immune escape ability. The CLSM images declared a lower uptake of MSF@CCM than that of MSF after incubation with RAW 264.7 (Fig. 3c). Compared to the MSF group, the green fluorescence intensity of the MSF@CCM group was decreased by 3.3-fold (Fig. 3d). Collectively, these results implied that CCM camouflaged MSF could be used as a bio-barrier-adaptable nanomedicine, resulting in effective homologous targeting and immune escape effects.

### Pharmacokinetic and homologous targeting of MSF@CCM in vivo

To evaluate the in vivo pharmacokinetics of MSF and MSF@CCM, we measured the Fe content in the blood of mice after injecting MSF and MSF@CCM through the tail vein. Mice injected with MSF were set as the control group. The MSF@CCM group with a higher Fe content exhibited significantly higher blood retention over a span of 48 h (Supplementary Fig. 13). Therefore, CCM-coated MSF exhibited superior blood retention, suggesting that the immune escape surface makeup of CCM had been successfully translocated onto the MSF surface. Based on the effective homologous targeting ability of MSF@CCM in vitro, the accumulation of MSF and MSF@CCM was further investigated in vivo. To ensure the MSF and MSF@CCM could be *i.v*., the hemolysis rate of MSF and MSF@CCM was first conducted. The hemolysis rate was less than 5% even at 200 μg mL^−1^, indicating that both MSF and MSF@CCM had good biosafety (Supplementary Fig. 14). Subsequently, the fluorescent dye indocyanine green (ICG) conjugated MSF (I-MSF) and MSF@CCM (I-MSF@CCM) were *i.v*. injected into 4T1 tumor-bearing mice. The results showed that MSF@CCM rapidly accumulated in tumor within 4 h, and exhibited a higher fluorescence signal in the tumor tissues than MSF (Supplementary Fig. 15). The fluorescence intensity of MSF@CCM was 2.0-fold higher than that of the MSF group, indicating that CCM camouflaged could improve the tumor targeting. Meanwhile, tumor and major organs (heart, liver, spleen, lung, and kidney) were acquired and imaged after 4 h administration. The results showed that MSF@CCM selectively accumulated at the tumor site (Supplementary Fig. 16). Furthermore, the tumor and major organs were excised after being injected different time (1, 4, 8, 12, 24, and 48 h) and the Fe content was detected by ICP-MS analysis, the results showed that MSF@CCM group accumulated 2.0-fold higher than that of MSF group at the tumor site at 4 h, which was consistent with the results of fluorescence imaging (Fig. 3e and Supplementary Fig. 17). Additionally, there was no significant accumulation of MSF@CCM in the major organs, indicating that MSF@CCM may maximize the therapeutic efficacy and alleviate non-target organ toxicities by clearing from major organs (e.g., liver). Moreover, Prussian blue staining of tissue slices, which could be labeled with iron ions, indicated that the accumulation of MSF@CCM in tumor tissues was much greater than that of MSF (Fig. 3f). These results further illustrated the coating of CCM could overcome the biological barrier and ultimately achieved targeted accumulation in the tumor tissues.

### Cavitation effect of MSF@CCM

**Fig. 4:**
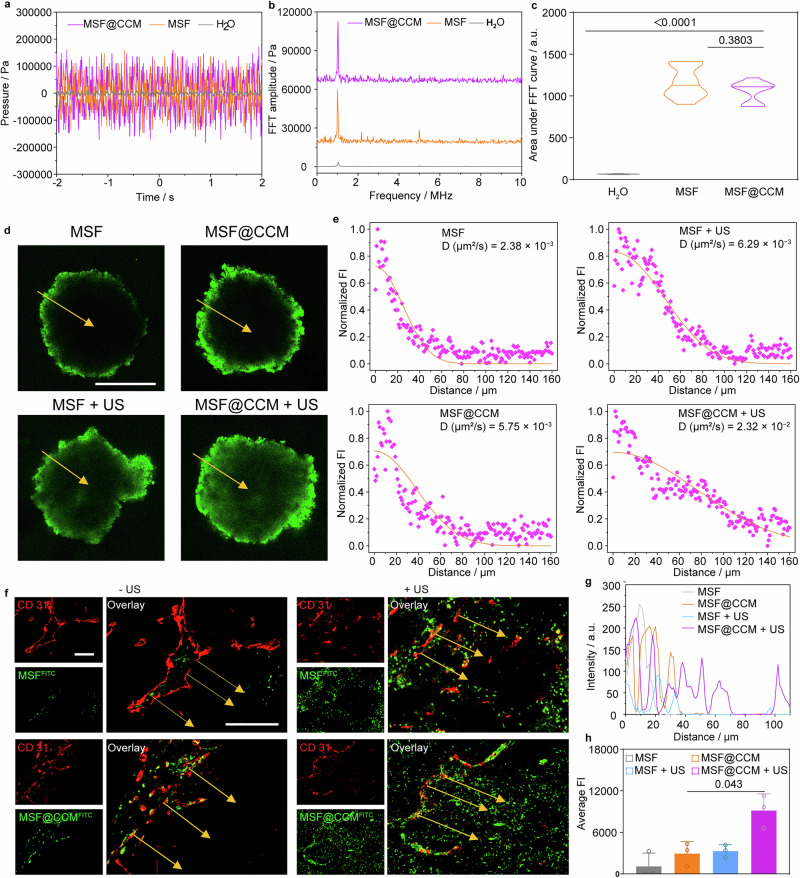
Cavitation effect and tumor penetration of MSF@CCM. **a** Noise signals obtained by the hydrophone for H_2_O, MSF and MSF@CCM groups. **b** FFT spectra of H_2_O, MSF and MSF@CCM cavitation noise signals obtained by hydrophone. **c** Integral areas under the FFT curves for different groups. Data are presented as mean ± SD (*n* = 10). **d** CLSM images of the tumor penetration of MSF and MSF@CCM in MTSs with or without US (1.0 MHz, 1 W cm^−2^, 3 min, 50% duty cycle) irradiation for 24 h. Scale bar = 200 μm. **e** The corresponding variance of fluorescence signal intensity along the dotted lines for 4T1 MTSs 24 h in (**d**). **f** The penetration of MSF and MSF@CCM after *i.v*. injection without or with US irradiation (1.0 MHz, 1.5 W cm^−2^, 5 min, 50% duty cycle) in the tumor. Blood vessels are labeled with CD31 (red), and MSF and MSF@CCM are labeled with FITC (green), respectively. Scale bar = 100 μm. **g** The corresponding fluorescence intensity of green across along the lines in the 4T1 tumor section images in (**f**). **h** Average fluorescence intensity from the blood vessel to the in-depth tumor parenchyma in the selected region at (**f**), as indicated by yellow arrows. Data are presented as mean ± SD (*n* = 3). One-way ANOVA with Tukey’s multiple comparison was used to calculate statistical differences in **c** and **h**

To investigate the cavitation effects of MSF and MSF@CCM, the cavitation noise signals of different groups (H_2_O, MSF, and MSF@CCM) under the same US irradiation conditions (1.0 MHz, 1.5 W cm^−2^, continuous) were measured by hydrophone. All solvents were degassed to eliminate the interference of dissolved gases in the solution on the cavitation effect. The results showed that the sound pressure amplitude of the MSF and MSF@CM groups was significantly higher than that of the control group, indicating that both MSF and MSF@CCM could effectively induce cavitation under US irradiation (Fig. 4a). However, the cavitation effect is a complex process. This is attributed to that the acoustic emission generated under the US exposure includes the ultrasonic transducer acoustic signal, stable cavitation, transient cavitation and background noise, which makes it difficult to obtain purely transient cavitation noise from the total signal.^39^ The Fast Fourier Transform (FFT) transforms the cavitation noise signal into a frequency amplitude spectrogram to identify the applied field fundamental frequency *f*_0_ (1 MHz), subharmonic spectrum *f*_0_/2, super harmonic spectrum 2*f*_0_, and the broadband noise spectrum (Fig. 4b).^40^ In particular, the super harmonic and broadband noise spectra were widely used to evaluate the cavitation intensity, which mainly originates from the energy release resulting from the collapse of transient cavitation bubbles.^41,42^ Therefore, 10 cycles of data collection and processing were conducted based on the assessment methods and principles that have been reported (Supplementary Figure 18).^43–45^ The results showed that the integrated areas of the MSF and MSF@CCM groups were significantly higher than that of the control group, but were not significantly different from each other (Fig. 4c). These results may be attributed to that the porous structure and rough surface of MSF provide stable nucleation sites for cavitation nucleation, while the CCM coating has no significant effect on the generation of cavitation effect. These effects ultimately lead to biological effects, such as sonoporation and ROS generation.

### US-enhanced penetration in vivo and in vitro

The multicellular tumor spheroids (MTSs) model has been widely used to evaluate the penetration and diffusion ability of nanomedicines in solid tumors because they could well simulate the in vivo tumor biological microenvironment.^46^ Moreover, US-induced mechanical force is an effective approach for drug delivery.^47,48^ Therefore, the penetration ability of MSF@CCM, labeled with FITC, with or without US irradiation into the tumor deep was further assessed on MTSs (Fig. 4d,e and Supplementary Fig. 19). First, MTSs were incubated with MSF and MSF@CCM for 4, 12, and 24 h, respectively. Meanwhile, the MSF + US and MSF@CCM + US groups utilized US treatment. Then, collected CLSM images of the MTSs with a scanning depth of 100 μm to investigate the diffusion profiles after different treatments. After 24 h incubation, compared to the MSF group, MSF@CCM showed stronger fluorescence intensity in the MTSs periphery, and the diffusion coefficient was 2.38 × 10^−3^ μm^2^ s^−1^ and 5.75 × 10^−3^ μm^2^ s^−1^, respectively. This result may be attributed to CCM-mediated homologous adhesion capacity, resulting in MSF@CCM could be more effectively internalized by 4T1 cells. Furthermore, after US irradiation treatment, the diffusion coefficient of MSF@CCM was 2.32 × 10^−2^ μm^2^ s^−1^, which was much higher than that of the MSF + US group (6.29 × 10^−3^ μm^2^ s^−1^). Compared to the MSF group, the MSF + US group showed a 2.6-fold increase in the diffusion coefficient. Compared to US treatment group, the only MSF and MSF@CCM groups possessed lower penetration and diffusion coefficients, attributed to their larger sizes,^49^ which were hindered by the dense cellular matrix formed by 4T1 cells. These results indicated that the US-induced cavitation effect could overcome the tumor interstitial barrier, allowing MSF@CCM to penetrate deep into the tumor effectively. To further validate the efficacy of US in promoting the penetration of MSF@CCM in vivo, we used fluorescent imaging to investigate the tumor distribution of FITC-labeled MSF and MSF@CCM, and the blood vessels were stained with red fluorescence (CD31). In the MSF@CCM + US group, tumor areas away from blood vessels showed a strong green fluorescent signal, which penetrated significantly deeper than in the MSF + US group (Fig. 4f, g). MSF was virtually impermeable to the interior of the tumor via blood vessels, whereas MSF@CCM was mainly restricted in the perivascular area of the tumor site. In contrast, after US treatment, a small proportion of MSF could enter the interior of the tumor, while majority of MSF@CCM could efficiently cross the vascular barrier and penetrate deep into the tumor parenchyma. MSF@CCM + US had 3.1-fold and 2.8-fold higher fluorescence signals than MSF@CCM and MSF + US (Fig. 4h), respectively. These results indicated that the MSF@CCM could efficiently accumulate in the tumor tissues and the US-triggered cavitation effect could further enhance the penetration of MSF@CCM deeper into the tumor.

### ROS generation and the mechanisms in vitro

**Fig. 5:**
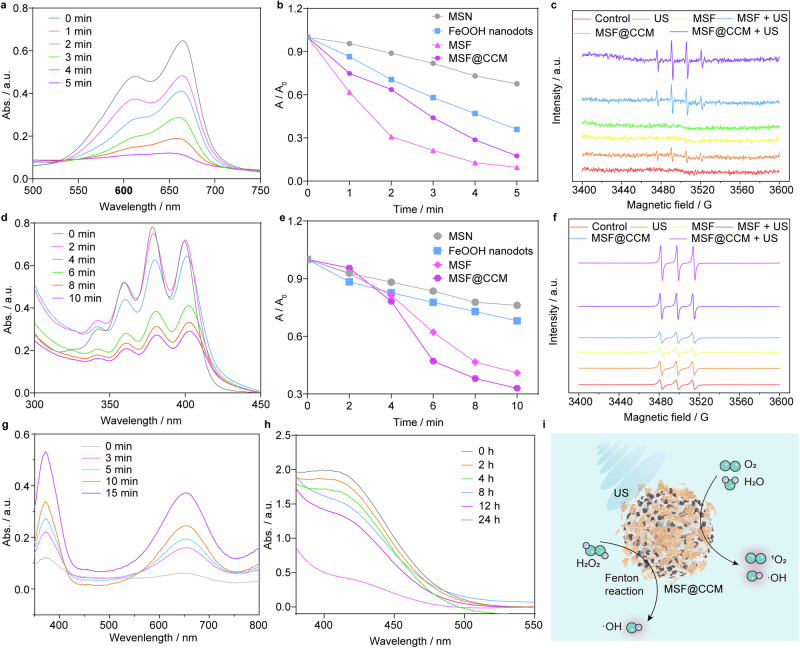
ROS generation of the as-synthesized MSF and MSF@CCM. **a** Time-dependent sono-degradation of MB caused by MSF@CCM. **b** The degradation rate of MB (A/A_0_) caused by MSN, FeOOH nanodots, MSF and MSF@CCM after US irradiation (1.0 MHz, 1.5 W cm^−2^). **c** ESR spectra of •OH after different treatments. US: 1.0 MHz, 1.5 W cm^−2^, 1 min. **d** Time-dependent sono-degradation of DPA caused by MSF@CCM. **e** The degradation rate of DPA (A/A_0_) caused by MSN, FeOOH nanodots, MSF and MSF@CCM after US irradiation (1.0 MHz, 1.5 W cm^−2^). **f** ESR spectra of ^1^O_2_ after different treatments. US: 1.0 MHz, 1.5 W cm^−2^, 1 min. **g** The UV−vis−NIR absorbance spectra of TMB after incubation with MSF@CCM for different times. **h** GSH depletion after incubation with MSF. **i** The proposed mechanism of ROS generation

Based on the superior targeting and penetration capabilities of this nanoplatform, the acoustic responsive activity of MSF@CCM and ROS generation capability was further validated by two commercial ROS detection probes, methylene blue (MB) and 9,10-diphenyl anthraquinone (DPA). The intensities of characteristic absorption peak of MB were gradually declined with the US irradiation time increased, indicating sustained hydroxyl radical (•OH) generation (Fig. 5a and Supplementary Fig. 20). As shown in Fig. 5b, after US irradiation, the content of MB in the MSF and MSF@CCM groups were decreased by 90.5% and 82.5%, respectively, while no obvious changes were observed in the FeOOH nanodots group and MSN group. Upon interaction with singlet oxygen (^1^O_2_), DPA could be oxidized to fluorescent 9,10-diphenyl anthraquinone (DPO_2_). Under the US trigger, MSF and MSF@CCM produced significantly higher levels of ^1^O_2_ in the solutions with a time-dependent manner, and the content of DPA were decreased by 59% and 67% in MSF and MSF@CCM groups, respectively, which were significantly higher than the MSN and FeOOH nanodots (Fig. 5d-e and Supplementary Fig. 21). Furthermore, the generation of •OH and ^1^O_2_ was further confirmed through the electron spin resonance (ESR) spectra. Compared to only the US group, the •OH production level in MSF and MSF@CCM increased by 113% and 186% under US irradiation, respectively (Fig. 5c and Supplementary Fig. 22a). Meanwhile, the ^1^O_2_ signal peaks were obviously observed in MSF + US and MSF@CCM + US groups, which were increased by 80% and 140%, respectively (Fig. 5f and Supplementary Fig. 22b). The introduction of porous MSN conferred a porous structure to the MSF and reduced the aggregation of FeOOH nanodots, thus improving the acoustic sensitivity. Meanwhile, availability of the porous structure significantly enhanced ROS production, which in turn showed great potential for eliminating cancer cells.^50^ Furthermore, the coating of CCM had a negligible effect on the generation of ROS. To further verify whether US affected CCM vesicles, the morphological changes of MSF@CCM and blank-CCM vesicles were evaluated by TEM after US irradiation. The results showed that blank-CCM vesicles had no visible changes before and after US irradiation, while an obvious membrane reduction was shown in the MSF@CCM + US group, indicating that US could disrupt cell membranes that were consistent with previous reports in the literature (Supplementary Fig. 23).^51^

The FeOOH nanodots were reported to have Fenton reaction activity.^52,53^ Therefore, we evaluated the ROS generation ability by 3,3′,5,5′-tetramethylbenzidine (TMB) assay. The results showed that MSF and MSF@CCM could effectively induce the generation of •OH by decomposition of H_2_O_2_ under acidic conditions (Fig. 5g and Supplementary Fig. 24). However, high glutathione (GSH) levels in cancer cells could scavenge excessive ROS, which would compromise the treatment efficiency.^54^ Thus, the GSH depletes ability of MSF was assessed by the 5,5’-Dithiobis-(2-nitrobenzoic acid) (DTNB). As shown in Fig. 5h, the absorbance of DTNB at ~412 nm decreased significantly with incubation time increased, indicating that MSF had a better GSH depletion capacity. Based on the above results, the possible mechanisms for ROS generation were proposed in Fig. 5i.

### Cytotoxicity and mechanisms in vitro

**Fig. 6:**
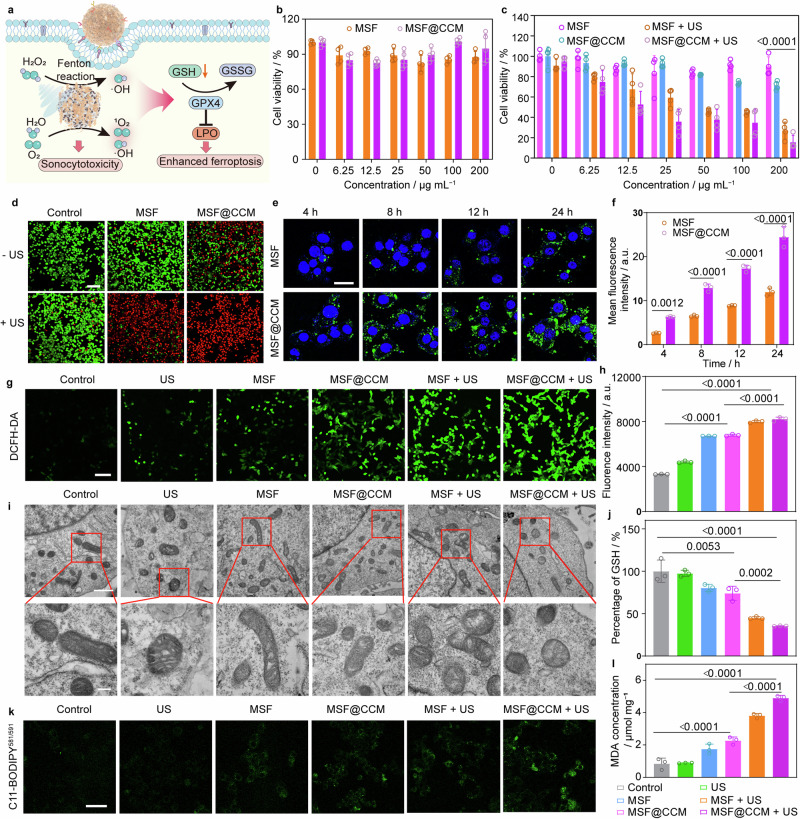
In vitro cytotoxicity and ferroptosis effect of MSF@CCM. **a** Schematic illustration of MSF@CCM-enhanced cytotoxicity and ferroptosis *via* producing ROS and LPO. **b** Cell viability of NIH-3T3 cells treated with MSF and MSF@CCM at different concentrations. Data are presented as mean ± SD (*n* = 4). **c** Cell viability of 4T1 cells treated with MSF and MSF@CCM at different concentrations with or without US irradiation. US: 1.0 MHz, 1.5 W cm^−2^, 2 min, 50% duty cycle. Data are presented as mean ± SD (*n* = 4). **d** CLSM images of 4T1 cells co-stained by Calcein AM/PI. Green (Calcein AM), live cells; red (PI), dead cells. US: 1.0 MHz, 1.5 W cm^−2^, 2 min, 50% duty cycle. Scale bar = 100 μm. **e** CLSM images of 4T1 cells incubated with FITC-labeled MSF and MSF@CCM for different time points. Scale bar = 25 μm. **f** Corresponding mean green fluorescence intensity of MSF and MSF@CCM in (**e**). Data are presented as mean ± SD (*n* = 3). **g** CLSM images and (**h**) corresponding fluorescence intensity of ROS with different treatments. Green (DCFH-DA), ROS. US: 1.0 MHz, 1.5 W cm^−2^, 2 min, 50% duty cycle. Scale bar = 100 μm. Data are presented as mean ± SD (*n* = 3). **i** Bio-TEM observation of mitochondrial morphology of 4T1 tumor cells after different treatment. Scale bar = 1 μm. Scale bar for localized magnification is 200 nm. **j** Intracellular GSH levels in 4T1 cells with different treatments. Data are presented as mean ± SD (*n* = 3). **k** CLSM images of cells stained by Liperfluo for monitoring intracellular LPO levels induced by different treatments. Scale bar = 50 μm. **l** Levels of MDA after different treatments. Data are presented as mean ± SD (*n* = 3). US: 1.0 MHz, 1.5 W cm^−2^, 2 min, 50% duty cycle. One-way ANOVA with Tukey’s multiple comparisons was used to calculate statistical differences in (**c**, **f**, **h**, **j**, and **l**)

Based on the previous better acoustic sensitization performance of MSF@CCM, the US-mediated cytotoxicity was further systemically investigated (Fig. 6a). The standard methyl thiazolyl tetrazolium (MTT) assay showed that MSF and MSF@CCM (200 μg mL^−1^) exhibited no obvious cytotoxicity after co-incubation 24 h with NIH-3T3 cells, indicating that MSF@CCM had negligible cytotoxicity within the concentration (Fig. 6b). When cultured with 4T1 cells, MSF@CCM showed dose-dependent cytotoxicity while MSF showed no obvious cytotoxicity (Fig. 6c). Note that MSF (24.1%) and MSF@CCM (12.6%) groups exhibited excellent potent US-triggered cell killing capability at a concentration of 200 μg mL^−1^, which might be attributed to the superior ROS generation capacity (Fig. 6c). Subsequently, the therapeutic effect of MSF@CCM with or without US were further validated by staining dead and live cells with propidium iodide (PI) and calcein acetoxymethyl ester (Calcein-AM), respectively (Fig. 6d and Supplementary Fig. 25). In the CLSM images, the control and US groups showed negligible cell death (almost green fluorescence), while MSF@CCM groups showed partial cell death (most green fluorescence), and the MSF + US and MSF@CCM + US groups appeared the most significant red fluorescence, indicating the excellent cytotoxicity of MSF@CCM after US irradiation.

Subsequently, the MSF@CCM-mediated mechanism of cytotoxicity was further validated. First, we observed the uptake of FITC-MSF and FITC-MSF@CCM after incubation with 4T1 cells (Fig. 6e and Supplementary Fig. 26). CLSM images showed that the green fluorescence signals of the FITC-MSF@CCM group was significantly increased in 4T1 cells compared to that in the FITC-MSF group, and the fluorescence intensity of FITC-MSF@CCM group was 2.4, 2.0, 2.0 and 2.0-folds that of FITC-MSF group after co-incubation of 4, 8, 12, and 24 h, respectively (Fig. 6f). These results indicated that CCM-coating could effectively enhance cell uptake. The intracellular ROS levels were further detected by 2, 7-dichlorofluorescein diacetate (DCFH-DA). As shown in Fig. 6g,h, a large amount of green fluorescence was observed in the MSF@CCM + US group compared to the control, US, and MSF groups, indicating that MSF@CCM could effectively generate ROS under US irradiation. Ferroptosis is a form of programmed cell death that is different from cell necrosis, apoptosis, and autophagy, involving GSH depletion, LPO accumulation, and other key parameters.^55^ Mitochondrial morphology was characterized by bio-transmission electron microscopy (bio-TEM) (Fig. 6i). Compared with the control group, the MSF@CCM + US group showed typical features of mitochondrial morphology such as mitochondrial atrophy and mitochondrial ridge loss, suggesting that MSF@CCM combined with US lead to the destruction and dysfunction of mitochondrial structure induced by ferroptosis. Moreover, compared with the control group, the MSF@CCM and MSF + US group also showed slight mitochondrial ridge loss, suggesting that effective internalization or stimulation by US could induce ferroptosis. 5,5,6,6-tetrachloro-1,1,3,3-tetraethylbenzimidazolylcar-bocya-nine iodide (JC-1) fluorescent probes was used to evaluate the changes of mitochondrial membrane potential (MMP) after different treatments. Compared with the MSF@CCM group, the MSF@CCM + US group showed more green fluorescence, indicating that the number of damaged mitochondria was increased, which was consistent with the results of bio-TEM (Supplementary Fig. 27). The GSH depletion at the cellular level was further verified. The MSF@CCM + US group displayed more intracellular GSH depletion than the MSF + US group (Fig. 6j). Besides, MSF@CCM irradiated by the US exhibited significant LPO accumulation and malondialdehyde (MDA) production (Fig. 6k,l). Meanwhile, the ferroptosis-related proteins expression of GPX4 and ACSL-4 was investigated by Western blotting analysis. In Supplementary Fig. 28, compared to other group, the protein expression levels of GPX4 and ACSL-4 in the MSF@CCM + US group were significantly reduced. These results indicated that the MSF@CCM combined with US irradiation effectively induces cellular ferroptosis in vitro. These results indicated the activation of ferroptosis pathways by ROS generation and GSH depletion, which was enhanced after US irradiation.

### Tumor suppression in vivo

**Fig. 7:**
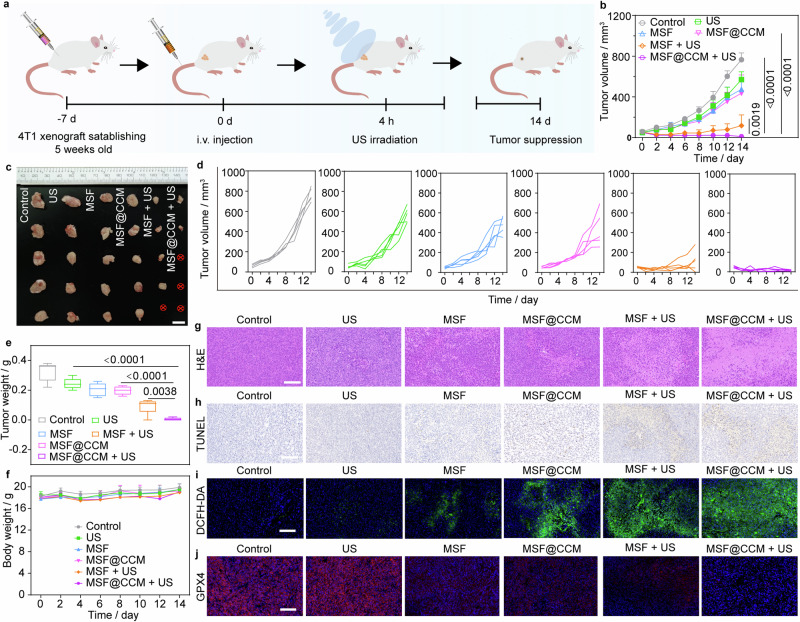
MSF/MSF@CCM-mediated cancer therapy in vivo. **a** Schematic illustration of MSF/MSF@CCM induced tumor suppression. **b** Tumor volume of mice with different treatments. **c** Photographs of isolated tumors in different groups. Scale bar = 1 cm. **d** Tumor volume of mice with different groups. **e** Relative tumor weight of different groups at day 14. **f** Curves of mice body weight with different treatments. **g** H&E-stained and (**h**) TUNEL staining of the tumors after different treatments. **i** Fluorescence images of tumor slices collected after treatment for 4 h and stained with DCFH-DA. **j** Immunofluorescence images of GPX4 expression in tumor tissues after different treatments (red: GPX4, blue: nuclei). Scale bar = 100 μm. US: 1.0 MHz, 1.5 W cm^−2^, 5 min, 50% duty cycle. Data are presented as mean ± SD (*n* = 5). One-way ANOVA with Tukey’s multiple comparison was used to calculate statistical differences in (**b**) and (**e**)

Motivated by the effective therapeutic effect of MSF@CCM-mediated cytotoxicity and ferroptosis in vitro, the antitumor efficacy of MSF@CCM combined with US irradiation in vivo was further conducted. The 4T1 tumor-bearing mice were randomly divided into six groups (*n* = 5): Control, US, MSF, MSF@CCM, MSF + US, and MSF@CCM + US. At 4 h post *i.v*. injection of MSF and MSF@CCM, the tumors were treated with US irradiation (Fig. 7a). During this treatment process, the tumor volume of the MSF@CCM group was inhibited compared to the control group (Fig. 7b–d). Meanwhile, MSF + US showed a desirable therapeutic effect (81.81%). Specifically, MSF@CCM + US showed the best therapeutic effect, and the tumor inhibition efficiency was about 96.50% (Fig. 7c–e). These efficient tumor inhibition rates were attributed to the effective tumor accumulation and US assistance. Importantly, the mouse body weights showed no significant change during the treatment period, indicating that US-mediated tumor management had a better biosafety profile (Fig. 7f). Besides, we evaluated the in vivo toxicity by blood biochemistry assay. We found that red blood cells (RBC), hemoglobin (HGB), hematocrit (HCT), mean corpuscular volume (MCV), mean corpuscular hemoglobin (MCH), mean corpuscular hemoglobin concentration (MCHC), and platelets (PLT) were at normal levels at the end of treatment and white blood cells (WBC) were significantly lower in the MSF@CCM + US group, attributed to reduced inflammation in vivo following tumor elimination (Supplementary Fig. 29).^56^ Moreover, hematoxylin and eosin (H&E) staining of major organs showed no significant toxicity observed, further demonstrating the good biocompatibility of US in combination with MSF@CCM-mediated cancer therapy (Supplementary Fig. 30).

To gain further insight into the mechanisms of cancer therapy in vivo, H&E, TdT-dependent dUTP-biotin nick end labeling (TUNEL), ROS, and GPX4 staining of tumor tissues were performed. The H&E staining results indicated that MSF and MSF@CCM could effectively induce apoptosis and necrosis of cancer cells with the assistance of US irradiation (Fig. 7g). Furthermore, Fig. 7h showed a significant positive apoptosis signal in the MSF + US and MSF@CCM + US groups compared to the MSF and MSF@CCM groups. The tumor damages were further attributed to the high levels of ROS generation in tumor slices, especially the MSF + US and MSF@CCM + US group, which showed strong green fluorescence signals (Fig. 7i and Supplementary Fig. 31). To explore the US-assisted MSF@CCM-mediated ferroptosis, the expression of GPX4 was monitored. Immunohistochemistry staining of tumor tissues showed an obvious downregulation of GPX4 for MSF + US and MSF@CCM + US groups, implying MSF and MSF@CCM could effectively induce ferroptosis and further enhanced with the assistance of US (Fig. 7j and Supplementary Fig. 32).

## Discussion

Nanomaterials offer unique physicochemical properties advantageous in nanomedicine, including high surface area, porosity, and amphiphilicity, leading to applications in drug delivery, imaging, and cancer therapy.^57^ Particularly in tumor therapy, nanomedicines have demonstrated the ability to enhance the accumulation at tumor sites through active or passive targeting mechanisms while minimizing off-target toxicity to normal tissues. Despite their promising therapeutic efficacy in experimental studies and preclinical models, the clinical translation of nanomedicines remains challenging, limiting their widespread application.^58^ Targeted accumulation of nanomedicines is one of the key factors that influence therapeutic efficacy. Although the EPR effect occupies a crucial role in passive tumor targeting, which is limited by tumor heterogeneity, vascularization status, interstitial pressure, and lymphatic drainage.^59^ These factors significantly reduce the clinical applicability of EPR-based passive targeting. Active targeting strategies, which involve surface modification of nanomedicines with specific ligands (e.g., antibodies, aptamers, or small molecules) to enhance the recognition of tumor cell surface receptors, are also limited by the high heterogeneity and dynamic expression of these receptors, leading to inconsistent targeting efficiency and suboptimal therapeutic efficacy.^60^ Additionally, the human immune systems, especially RES, is able to rapidly recognize and phagocytose exogenous nanomedicines, leading to a shortened half-life of nanomedicines in the blood circulation and reducing their accumulation at tumor site.^61^ Meanwhile, physical barriers in solid tumors, including ECM, elevated interstitial fluid pressure, and poor tumor perfusion, further hinder the deep penetration of nanomedicines, thereby limiting their therapeutic efficacy.^62^ Furthermore, hypoxia and immunosuppressive tumor microenvironments can further exacerbate these challenges by promoting drug resistance and limiting the efficacy of nanomedicine-mediated therapies. Therefore, improving the tumor-specific accumulation, deep tissue penetration, and in vivo stability of nanomedicines remains an urgent challenge in the field of nanomedicine.

In recent years, biomimetic nanotechnology, leveraging cell membranes to camouflage nanomedicines, has emerged as a powerful strategy to overcome these limitations by enhancing targeting, prolonging circulation, and improving therapeutic efficacy.^63^ This approach exploits inherent biological functions, including homotypic targeting and immune evasion, representing a significant step toward precision medicine. Recent efforts have further advanced biomimetic drug delivery by engineering cell membrane-derived vesicles for targeted payload delivery and modulation of specific signaling pathways. For example, Liao et al. propose an efficient strategy for lesion-pathogen dual-targeting in the management of tuberculosis via camouflaging polymer cores with *mycobacterium*-stimulated macrophage membranes.^64^ Moon et al. report pathogen-mimicking hollow nanomedicines of mannan to limit the proportion of regulatory T cells and induce T_H_17 cell-mediated antitumor immune responses.^65^ This highlights the growing potential of biomimetic strategies to enhance therapeutic efficacy by precisely targeting diseased cells and manipulating intracellular signaling. Additionally, hybrid biomimetic nanoplatforms that integrate multiple cell membrane sources, such as combinations of tumor cell membranes and immune cell membranes, have demonstrated potential for immune microenvironment modulation and enhanced tumor accumulation.^16^ US, with its excellent tissue penetration ability and non-invasiveness, has been widely utilized in drug delivery and tumor therapy. US-mediated tumor therapy employs multiple mechanisms to induce cancer cell death, including direct activation of sonocatalytic nanomedicines to generate ROS, induce cavitation effects, and mechanical disruption.^17^ The cavitation effect transiently increases vascular and cellular membrane permeability, disrupting the dense ECM, and thereby improving the intratumoral distribution of nanomedicines.^18^ Additionally, this effect enhances cellular uptake and deep tumor penetration, leading to greater drug retention at the tumor site and improved therapeutic efficacy.^66^ Given these advantages, the integration of US technology with advanced nanomedicine platforms holds great promise for enhancing drug delivery efficiency, overcoming biological barriers, and boosting cancer treatment, offering a prospective strategy for precision tumor therapy.

Building upon these advances, we developed MSF@CCM, a bio-barriers adaptable biomimetic nanoplatform, designed to overcome the challenges of tumor targeting and penetration while amplifying therapeutic efficacy through ferroptosis induction. By integrating the homotypic targeting effects of CCM camouflaging with US-enhanced intratumoral penetration, MSF@CCM achieved a 2.0-fold increase in tumor accumulation and a 3.1-fold increase in penetration depth. More importantly, the mesoporous structure of MSF and its FeOOH composition confers superior ROS-generating capacity. Upon US stimulation, MSF@CCM significantly amplifies intracellular oxidative stress through cavitation effects and Fenton reactions, leading to LPO accumulation, mitochondrial morphological alterations, and downregulation of ferroptosis-associated proteins such as GPX4 and ACSL-4. These molecular events effectively induce cell death and suppress tumor growth. Compared with conventional sonocatalytic nanomedicines that rely solely on ROS generation, MSF@CCM exploits US-enhanced drug delivery combined with Fenton reaction-mediated ferroptosis amplification, providing a novel synergistic therapeutic strategy for cancer treatment. Furthermore, the incorporation of US-responsive components enables spatiotemporal control of sonocatalytic nanomedicines activation, potentially reducing systemic toxicity and improving treatment selectivity.

This work contributes to the growing body of evidence demonstrating the potential of cell membrane-coated nanomedicines to overcome biological barriers and manipulate intracellular signaling pathways for therapeutic benefit. By integrating multifunctional nanomedicines with the inherent biological properties of cell membranes, we can fine-tune interactions with the tumor microenvironment and enhance therapeutic outcomes. Looking ahead, the inherent bioadaptive properties of biomimetic nanomedicines hold substantial promise for developing targeted therapies against tumors with highly restrictive barriers, such as glioblastoma and pancreatic cancer. Moreover, the integration of biomimetic nanomedicines with immunotherapy, gene therapy, or engineered cell membrane modifications could further enhance tumor immune responses and improve targeting specificity, facilitating the development of next-generation precision nanomedicines. Further, the combination of US with immune checkpoint inhibitors or other immunomodulatory drugs may also have provided synergistic effects, resulting in a more effective anti-tumor immune response.

Thus, we developed MSF@CCM, a bio-barrier-adaptable nanomedicine that demonstrates a novel mechanism of action whereby the combination of biomimetic targeting, US-mediated penetration enhancement, and ROS-induced ferroptosis synergistically enhances antitumor activity. Our in vitro and in vivo results demonstrate that MSF@CCM, in combination with US, significantly inhibits tumor growth, achieving a tumor suppression rate of 96.5%, while maintaining excellent biocompatibility and minimal systemic toxicity. As biomimetic nanotechnology and physical energy-mediated activation strategies continue to evolve, MSF@CCM holds significant potential for clinical translation in treating aggressive and refractory tumors. Future, through in-depth exploration of its molecular mechanism and optimization of engineering design, constructing clinically relevant tumor models to further enhance the clinical translational value of US combined with biomimetic technology, ultimately promote the clinical translation of nanomedicine-based cancer therapy.

## Materials and methods

### Materials

FeCl_3_•6H_2_O, 9,10-diphenylanthracene (DPA), NH_4_HCO_3_, and methylene blue (MB) were obtained from Macklin. DMEM medium, fetal bovine serum (FBS), and PBS were obtained from Coning. 3-(4,5-dimethylthiazol-2-yl)-2,5-diphenyltetra-zolium bromide (MTT), 2,7-dichlorodihydrofluorescein diacetate (DCFH-DA), calcein acetoxymethyl ester and propidium iodide (Calcein-AM/PI) were purchased from Beijing Solarbio Science &Technology (China). Triethanolamine (TEA), cetyltrimethylammonium chloride (CTAC), N, N-Dimethylformamide (DMF) and (3-aminopropyl) triethoxysilane (APTES) were purchased from Aladdin. Tetraethyl orthosilicate (TEOS) and cyclohexane were purchased from Shanghai Macklin Biochemical Technology Co., Ltd. All reagents are used directly.

### Preparation of MSN

Three-dimensional dendritic MSN was synthesized according to the previous method.^67^ In detail, TEA (0.36 g), TCAC (48 mL) aqueous solution (25 wt%), and deionized water (72 mL) were added into a round-bottom flask and preheated for 1 hour at 60 °C with gentle stirring. Subsequently, a solution containing TEOS (4 mL) and cyclohexane (36 mL) was slowly transferred to the above-preheated solution and stirred 17 h at 60 °C. Centrifugation and washing to obtain the white solid. Then, the CTAC was removed by calcination at 550 °C for 2 h.

### Preparation of MSF

MSF was synthesized by in-situ growth. First, 1 mM of FeCl_3_•6H_2_O was dissolved in 40 mL of ethanol and then added 40 mg MSN, ultrasonic treatment 10 min. Then, 3 mM of NH_4_HCO_3_ was added to the mixture and continuously stirring 16 h. Centrifugation and obtained a yellow solid. To prepare FeOOH nanodots, 1 mM of FeCl_3_•6H_2_O was dissolved in 40 mL of ethanol and then added 3 mM of NH_4_HCO_3_. Continuous stirring 16 h and obtained products by centrifugation and washing.

### Cell culture

Mouse embryonic cells (NIH-3T3), human umbilical vein endothelial cells (HUVEC), squamous cell carcinoma (SCC), human lung cancer cells (A549), human breast cancer cells (MDA-MB-231), RAW 264.7 and 4T1 cells were cultured in the medium of Dulbecco’s modified Eagle medium (DMEM) containing 10% FBS and 1% penicillin/streptomycin. The cell lines were cultured in an incubator at 37 °C and containing 5% CO_2_.

### Preparation of cancer cell membrane and MSF@CCM

First, CCM was obtained by the extrusion method as previously reported. Briefly, 4T1 cells were collected by batch culture. The collected cells were dispersed in hypotonic Tris buffer (pH = 7.4), while PMSF was added and stored at 4 °C overnight. The cells were disrupted by extruding the 400 nm and 200 nm polycarbonate membranes back and forth 11 times. Afterwards, cell contents and organelles were then removed by low-speed centrifugation (×2000 *g*). The supernatant was collected and centrifuged at high-speed centrifugation (×20,000 *g*) to obtain cell membrane (CCM) sediment. Store at −80 °C for further use. To prepare MSF@CCM, MSF was mixed with CCM and then extruded through 400 nm and 200 nm polycarbonate membranes. MSF@CCM was collected by centrifugation. The particle size and zeta potentials of MSN, MSF, MSF@CCM, and CCM were measured by Dynamic light scattering (DLS).

### Characterization

The morphology and structure of mesoporous silica nanoparticle (MSN), MSF, and MSF@CCM were characterized by HT-7700 transmission electron microscope (TEM). JEM-2100F high-resolution TEM (JEOL, Japan) was applied to characterize MSF morphology and analyze the distribution of O, Si, and Fe. DLS was used to measure the sizes of MSN, MSF, and MSF@CCM. The crystal structure of MSF was characterized by powder X-ray diffraction (XRD) (XRD-6000, Japan). The specific surface area, pore diameter, and pore volume of MSF were measured by N_2_ adsorption-desorption system (ASIQK4000-5, America). ^1^O_2_ and •OH were quantified by an electron spin resonance (ESR) spectrometer (Bruker EMXplus). X-ray photoelectron spectroscopy (XPS) was used to evaluate the chemical state of Fe and O in MSF. DJO-2776 sonicator as an energy converter and was applied to treatment in vitro and in vivo.

### Cavitation effect of MSF@CCM

Specifically, all samples were experimented on DJO-2776 sonicator at 1 MHz. The acoustic signals of different groups (H_2_O, MSF, and MSF@CCM) were recorded with an MO hydrophone. In each experiment, the acoustic signals (generated by the cavitation phenomenon) were recorded every 0.02 seconds for 4 seconds, for a total of 10 cycles. The frequency component of the stimulation was removed by calculating the acoustic signal for each cycle by fast Fourier transform. Finally, the area under the curve of broadband noise (3.25*f*_0_-3.75*f*_0_) was calculated for each spectrum. This area was proportional to the energy released when the cavitation bubble collapsed.

### Characterization of cell membrane proteins

Sodium dodecyl sulfate-polyacrylamide gel electrophoresis (SDS-PAGE) was used to further examine the cell membrane proteins. The protein concentrations of CCM and MSF@CCM were quantified with the BCA assay kit. After being denatured, 15 μg of the specimen was added into a 15% SDS-PAGE and ran at 80 V for 30 min, followed by 150 V for 50 min. Finally, the obtained gel was stained with Coomassie blue, the gel was washed with decolorizing solution and imaged with the camera.

### Western blotting analysis

The membrane proteins were examined by Western blotting. Equal amounts of protein were isolated by SDS-PAGE gel and transferred to PVDF membranes. The membranes were then treated with 5% bovine serum albumin (BSA) in TBST buffer for 1 h. The membranes were labeled with an anti-CD44 (abcam, ab157107, dilutio1:2000) or anti-CD47 antibody (abcam, ab214453, dilutio1:2000) and incubated at 4 °C overnight. After rinsing with TBST buffer, the membrane was treated with HRP-conjugated goat anti-mouse IgG (secondary antibody) for 1 h. Protein bands were visualized by an enhanced chemiluminescence system (Pierce, USA). To measure the expression of the GPX4 (abcam, ab252833, dilutio1:2000) and ACSL-4 (proteintech, 22401-1-AP, dilutio1:1000) proteins, 4T1 cells (2×10^5^ cells/well) were seeded in a 6-well plate and cultured overnight. Afterward, the cells were incubated with DMEM, MSF, and MSF@CCM (200 μg mL^−1^) for 12 h and US irradiation (1.0 MHz, 1.5 W cm^−2^, 2 min, 50% duty cycle), continued cultured for another 12 h. Finally, the cells were collected and lysed with RIPA lysate containing 1% PMSF to extract total proteins and quantify the proteins with the BCA kit. Subsequent steps with reference to the methodology described above.

### Preparation of DiD-CCM

CCM was obtained by extrusion and density gradient centrifugation, dispersed in PBS, then DiD solution was added and incubated at 4 °C for 4 h avoiding light, and density gradient centrifugation was performed to obtain DiD-CCM.

### Preparation of FITC-MSF and FITC-MSF@CCM

10 mg of MSF was dissolved in DMF, dispersed by sonication, 0.3 mL of APTES was added at 300 rpm, stirred for 24 h without light, and washed by centrifugation to obtain MSF-NH_2_. MSF-NH_2_ was activated with NHS and EDC for 1 h, and then FITC was added and stirred for 24 h at 4 °C without light. Centrifugal washing was performed to obtain FITC-MSF, which was stored at a low temperature for further use. FITC-MSF@CCM was prepared by extruding CCM and FITC-MSF through polycarbonate membrane.

### In vitro homologous targeting ability of MSF@CCM

HUVEC, NIH-3T3, SCC, A549, MDA-MB-231, and 4T1 were seeded on CLSM-exclusive culture disk and allowed to adhere overnight, respectively. The FITC-MSF@CCM (100 μg mL^−1^) were added into cells and incubated for another 12 h. After this time, the cells were washed three times with PBS and observed by CLSM.

### Comparison of the RAW 264.7 cellular uptake of MSF and MSF@CCM

RAW 264.7 cells were cultured in CLSM-exclusive culture disk for 24 h. FITC-MSF and FITC-MSF@CCM were added into cells and incubated for another 12 h. Afterward, the cells were washed three times with PBS and observed by CLSM.

### In vitro MTSs penetration and treatment experiments

MTSs were cultured based on previous literature. 4T1 cells suspension (1000 per well) were grown in ultralow adsorption 96-well plates with 200 μL of medium, then changed every other day and incubated until 300 μm cell spheres were formed, followed by different treatments: (i) MSF, (ii) MSF + US, (iii) MSF@CCM, iv) MSF@CCM + US with a concentration of 200 μg mL^−1^ (US: 1.0 MHz, 1 W cm^−2^, 50% duty cycle, 3 min). The different treatments were followed by incubation for 4 h, 12 h, and 24 h, respectively. The cell spheres were then transferred to the CLSM dish by pipette, and finally the green fluorescence of the cell spheres was observed by CLSM. The diffusion coefficient was calculated by referring to previous literature and was calculated by normalizing the fluorescence intensity.^68^

### Tumor model

All animal experiments were carried out under the guidelines of the Animal Ethical and Welfare Committee of China-Japan Friendship Hospital (Permit Number zryhyy21-21-01-10). Female SPF BALB/c mice (6 weeks) were purchased from Beijing Vital River Laboratory Animal Technology Co., Ltd. The 4T1 tumor model was established in female SPF BALB/c mice.

### Hemolysis rate

The red blood cells were collected from BALB/c mice to evaluate the biocompatibility of MSF and MSF@CCM in vitro. First, the red blood cells were collected by centrifugation and washed with PBS three times to remove the white cells. Subsequently, the MSF and MSF@CCM were dispersed in PBS with a series concentration, followed by adding into the red blood cells, respectively. Simultaneously, water and PBS were used as positive and negative groups, respectively. The mixture was kept standing at room temperature for 4 h and collected by centrifugation at 3000 rpm for 10 min. The supernatant was collected and measured the absorbance at 570 nm. The hemolysis ratio was calculated by the following formula: hemolysis rate (%) = (sample absorption − negative control absorption)/(positive control absorption − negative control absorption) × 100%.

### Preparation of I-MSF and I-MSF@CCM

10 mg of MSF was dissolved in DMF, dispersed by sonication, 0.3 mL of APTES was added at 300 rpm, stirred for 24 h without light, and washed by centrifugation to obtain MSF-NH_2_. MSF-NH_2_ was activated with NHS and EDC for 1 h, and then ICG-COOH was added, and stirred for 24 h at 4 °C without light. Centrifugation and washing were performed to obtain ICG-MSF (I-MSF), which was stored at low temperatures for further use. I-MSF@CCM was prepared by extruding CCM and I-MSF through a polycarbonate membrane.

### In vivo biodistribution

4T1 tumor-bearing mice were i.v. injected with I-MSF and I-MSF@CCM (100 μL, 2 mg mL^−1^) and free ICG to monitor the in vivo behaviors *via* IVIS Spectrum Imaging System (PerkinElmer, Waltham, MA, USA). In addition, the region of interest (ROI) of the IVIS Spectrum Imaging System was used to quantify the fluorescence intensity of tumor tissues. In addition, the collected tumor and major organs (liver, kidney, heart, lung, and spleen) were weighted at 1, 4, 8, 12, 24, and 48 h post-injection, then dissolved in a solution containing 3:1 (v: v) hydrogen chloride and nitric acid, and the contents of Fe in tumor and major organs were analyzed.

### Prussian blue staining

4T1 tumor-bearing mice were *i.v*. injected with MSF and MSF@CCM (100 μL, 2 mg mL^−1^). After *i.v*. injected 4 h, the tumors were collected and stained with Prussian blue to confirm the Fe accumulation in tumor tissues.

### In vivo penetration

4T1 tumor-bearing mice were used to investigate US-mediated penetration in vivo. FITC-labeled MSF and MSF@CCM (2 mg mL^−1^, 100 μL) were injected via *i.v*. injection, respectively. 4 h after injection, the MSF + US and MSF@CCM + US groups were processed for US (1.0 MHz, 1 W cm^−2^, 50% duty cycle, 5 min) treatment and tumor tissues were collected after US treatment 1 h, then sections were stained by CD31 to observe the penetration of MSF and MSF@CCM at the tumor sites.

### Detection and assessment of ^1^O_2_

MSF@CCM (100 μg mL^−1^) were mixed with DPA (1 mg mL^−1^). Subsequently, the degradation curve of DPA by MSF@CCM under US irradiation and recorded by UV−vis−NIR spectrophotometer. As comparison, the DPA degradation curve of MSN, FeOOH nanodots, and MSF were also recorded. Simultaneously, the change absorbance was used to quantify the degradation rate.

### Quantitative analysis of the generation of •OH

100 μg mL^−1^ of MSF@CCM were mixed with 5 μg mL^−1^ of MB. After US irradiation, the absorbance of MB was recorded by UV−vis−NIR spectrophotometer. As comparison, the MB degradation curve of MSN, FeOOH nanodots, and MSF were further recorded. The change absorbance was used to quantify the degradation rate.

### ESR measurements

^1^O_2_ and •OH were quantified by ESR using TEMP and DMPO as the trapping agent, respectively. Briefly, 3 μL of TEMP was added into 100 μL of nanomedicines solutions with a concentration of 100 μg mL^−1^ and with or without US irradiation. 10 μL of DMPO was added into 50 μL of nanomedicines solutions with a concentration of 100 μg mL^−1^and with or without US irradiation. All the experiments were carried out at room temperature.

### GSH depletion

MSF (200 μg mL^−1^) was incubated with GSH (50 mM) for different times (0, 1, 4, 8, 12, 24 h). Subsequently, the chromogenic agent DTNB (12.5 mM) was added, and the absorbance changes at 412 nm were measured by UV–vis–NIR.

### In vitro sonocytotoxicity of MSF and MSF@CCM

4T1 cells (1 × 10^4^ cells) were cultured in 96-well plate for 24 h. Then different concentrations (0, 6.25, 12.5, 25, 50, 100 and 200 μg mL^−1^) of MSF and MSF@CCM with fresh DMEM were added into the 96-well plate and cultured for another 24 h. Subsequently, MTT assay was used to detect the cell viabilities. For in vitro sonocytotoxicity experiments, 4T1 cells were cultured in 96-well plate for 24 h. Then MSF and MSF@CCM was dispersed in fresh DMEM and diluted to a series of concentrations (0, 6.25, 12.5, 25, 50, 100 and 200 μg mL^−1^). Subsequently, these DHMS solution was added to the 96-well plate and cultured for 12 h. After that, the cells were irradiated by US (1.0 MHz, 1.5 W cm^−2^, 2 min, 50% duty cycle) and cultured for another 12 h. Finally, in vitro US-mediated kill efficiency was evaluated by MTT assay.

### Co-staining of 4T1 cells with Calcein-AM and PI

4T1 cells were cultured in CLSM-exclusive culture disk for 24 h. Then, the old medium was discarded and the fresh medium of DMEM with a concentration of 200 μg mL^−1^ MSF@CCM was for incubated 12 h, followed by US irradiation (1.0 MHz, 1.5 W cm^−2^, 2 min, 50% duty cycle). Then, PI and Calcein-AM staining the cells at 37 °C for 30 min. Finally, the cells were washed three times with PBS and observed by CLSM. For comparison, the same procedure was used in the cells group, MSF group, MSF@CCM group, US group, and MSF + US group.

### Cell level ROS assay

To detect the ROS generation in 4T1, the CLSM was used to observe it. 4T1 cells were cultured in CLSM-exclusive culture disk for 24 h. MSF and MSF@CCM (100 μg mL^−1^) were added into 4T1 cells and incubated for another 12 h. Then, followed by US (1.0 MHz, 1.5 W cm^−2^, 2 min, 50% duty cycle) irradiation and stained with DCFH-DA for 30 min. Finally, the ROS fluorescence signal was detected by CLSM. Simultaneously, we set up cells-only control, US, MSF, MSF@CCM, and MSF + US group as the comparison. These groups adopted the same procedure as MSF@CCM group.

### LPO accumulation

Cells (20 × 10^4^ per well) were seeded in CLSM-exclusive culture disk and cultured for 24 h. The old DMEM was replaced with either pure DMEM or DMEM containing MSF (200 μg mL^−1^) or MSF@CCM (200 μg mL^−1^), with two replicates per group, incubated for another 12 h. One replicate was subjected to US treatment (1.0 MHz, 1.5 W cm^−2^, 50% duty cycle, 2 min). After further incubation for 12 h, the cells were stained according to the Liperfluo protocol and observed by CLSM.

### Comparison of the 4T1 cellular uptake of MSF and MSF@CCM

4T1 cells were cultured in a CLSM-exclusive culture disk and allowed to adhere overnight. FITC-MSF and FITC-MSF@CCM were added to cells and incubated for different time to observe the uptake by CLSM.

### In vivo tumor suppression

The 4T1 tumor-bearing mice were allocated into six groups (n = 5 each group): (i) control group with only PBS injection; (ii) US group; (iii) MSF group (MSF *i.v*. injection, 10 mg kg^−1^); (iv) MSF@CCM (MSF@CCM *i.v*. injection, 10 mg kg^−1^); (v) MSF + US (MSF *i.v*. injection, 10 mg kg^−1^) and (vi) MSF@CCM + US group (MSF@CCM *i.v*. injection, 10 mg kg^−1^). At 4 h after *i.v*. injection, the tumors were treated with US irradiation (1.0 MHz, 1.5 W cm^−2^, 5 min, 50% duty cycle). The treatment lasted for 14 days. Body weights and tumor sizes were monitored every two days. The volume of the tumor was calculated by the following equation: volume = width^2^ × length/2. End of treatment, all the mice were sacrificed, collected the tumors and major organs for immunohistochemistry staining to analyze the therapeutic mechanism and biocompatibility.

### Pharmacokinetic studies

To study the pharmacokinetics of MSF and MSF@CCM, 6 BALB/C mice (female, 4–6 weeks) were injected with 100 μL PBS (10 mg kg^−1^) containing MSF and MSF@CCM. 50 μL blood was collected from the retro-orbital of the mouse at different time points (0, 0.25, 0.5, 1, 2, 4, 8, 12, 24, 48 h) after injection. Aqua regia (Hydrochloric acid: Nitric acid = 1: 3) was dissolved for 24 h, and then heated in an oil bath at 70 °C for 24 h until a clear solution was obtained, filtered, and diluted, quantified samples for Fe content with ICP-MS.

### GPX4 immunofluorescence

After treatment, the obtained isolated tumors were sliced, paraffin-embedded, dewaxed, washed, and subjected to antigen repair. Excessive evaporation of buffer should be prevented during the repair process and avoid drying out the slides. After repaired, cool naturally. The slides were then washed three times with PBS (pH 7.4) on a decolorizing shaker, 5 min each time. After slight drying, the tissue sections were circled with a histochemical pen, and BSA was added for blocking for 30 min. Anti-GPX4 was added, and the slides were incubated overnight at 4 °C in a humidified chamber. The slides were then washed three times with PBS (pH 7.4) on a decolorizing shaker, 5 min each time. Secondary antibodies were applied and incubated in the dark at room temperature for 50 min. After washing three times with PBS (pH 7.4) on a decolorizing shaker, 5 min each time, the nuclei were stained with DAPI and incubated in the dark at room temperature for 10 min. The slides were washed three times with PBS (pH 7.4) on a decolorizing shaker, 5 min each time. Self-fluorescence quencher was added for 5 min, followed by rinsing with water for 10 min. The slides were then mounted, and images were acquired.

### Blood biochemistry analysis

The blood samples were collected at the end of treatment and used to conduct the blood biochemistry assay.

### Statistical analysis

All data were shown as mean ± standard deviation or mean. The statistical analysis was calculated by one-way analysis of variance (ANOVA) with Tukey post-hoc test.

## Supplementary information

Revised Supplementary Information

## Data Availability

All data generated or analyzed during this study have been included either in this article or in the supplementary information files.
