# Supplementary material for: Bio-barrier-adaptable biomimetic nanomedicines combined with ultrasound for enhanced cancer therapy
Source: Signal Transduct Target Ther. 2025 Apr 25;10:137. doi: 10.1038/s41392-025-02217-8 (PMC12022184; doi:10.1038/s41392-025-02217-8)
Supplement: Supplementary file 1 — Revised Supplementary Information [file 41392_2025_2217_MOESM1_ESM.docx]

Supplementary Materials for

**Bio-barrier-adaptable biomimetic nanomedicines combined with ultrasound for enhanced cancer therapy**

Juan Guo^1#^, Xueting Pan^1#^, Qingyuan Wu^1^, Ping Li^2^, Chaohui Wang^1^, Shuang Liu^1^, Haoyuan Zhang^1^, Zezhong Huang^1^, Xiaozhou Mou^2*^, Huiyu Liu^1*^, Jiajia Xue^1*^

Correspondence to: mouxz@zju.edu.cn, liuhy@mail.buct.edu.cn or jiajiaxue@mail.buct.edu.cn

**This PDF file includes:**

Figures. S1 to S32

**Other Supplementary Materials for this manuscript include the following:**

No other supplementary materials

**Figure S1.**


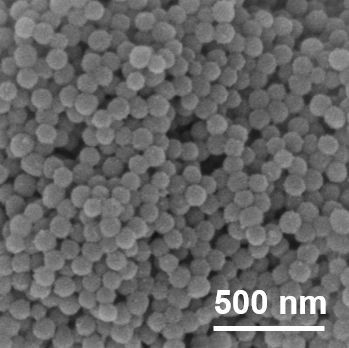


**Supplementary Figure 1.** SEM image of MSF.

**Figure S2.**

**
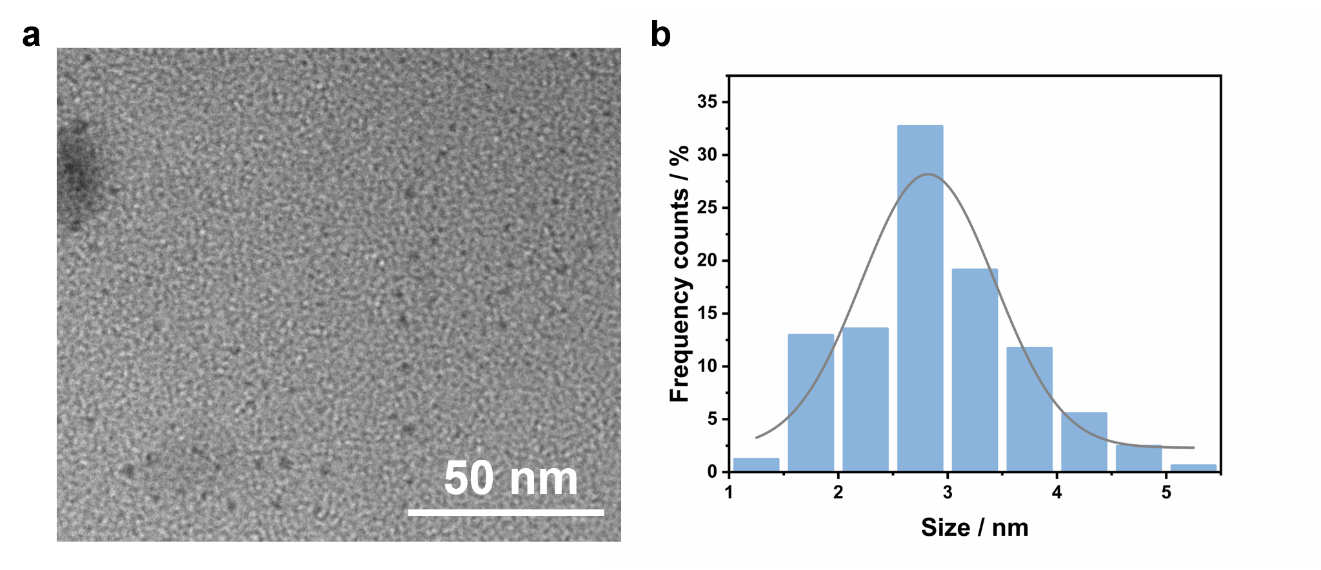
**

**Supplementary Figure 2. a**) TEM image of FeOOH nanodots and **b**) the size distribution of FeOOH nanodots.

**Figure S3.**

**
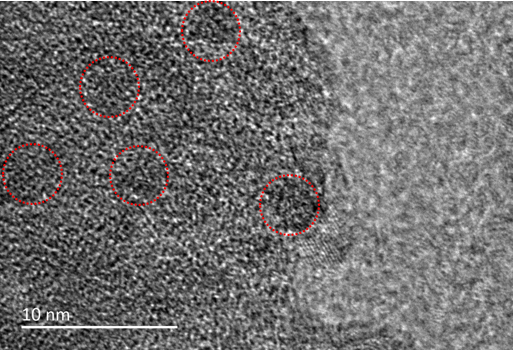
**

**Supplementary Figure 3.** HR-TEM of MSF. FeOOH nanodots are marked with red circle.

**Figure S4.**

**
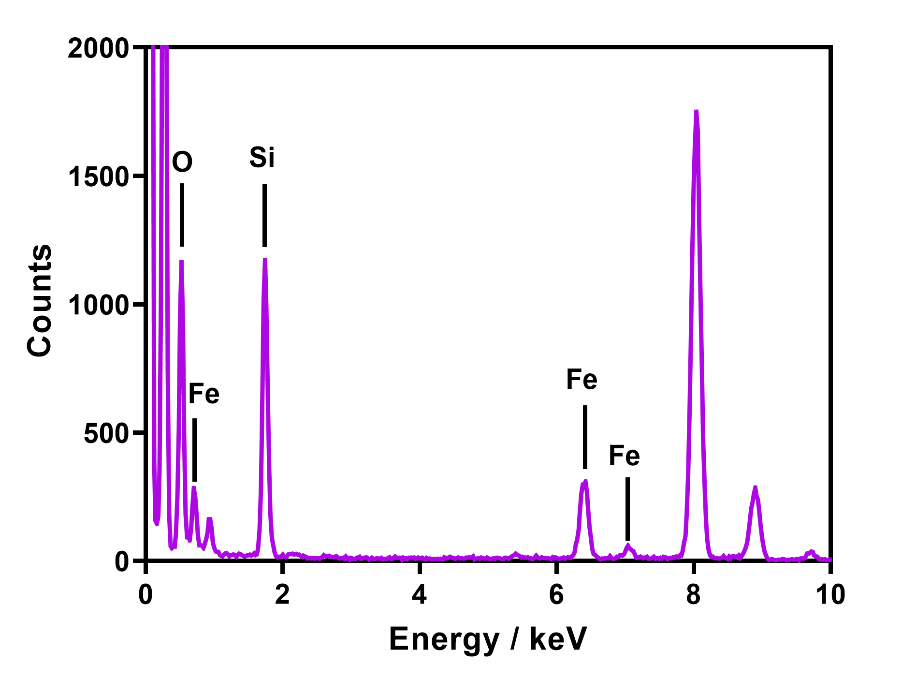
**

**Supplementary Figure 4.** EDS spectrum of MSF.

**Figure S5.**





**Supplementary Figure 5.** Pore size distribution curves of MSN and MSF.

**Figure S6.**

**

**

**Supplementary Figure 6.** XRD patterns of MSF, MSN and FeOOH nanodots.

**Figure S7.**

**

**

**Supplementary Figure 7.** FTIR spectra of FeOOH nanodots, MSF and MSN.

**Figure S8.**


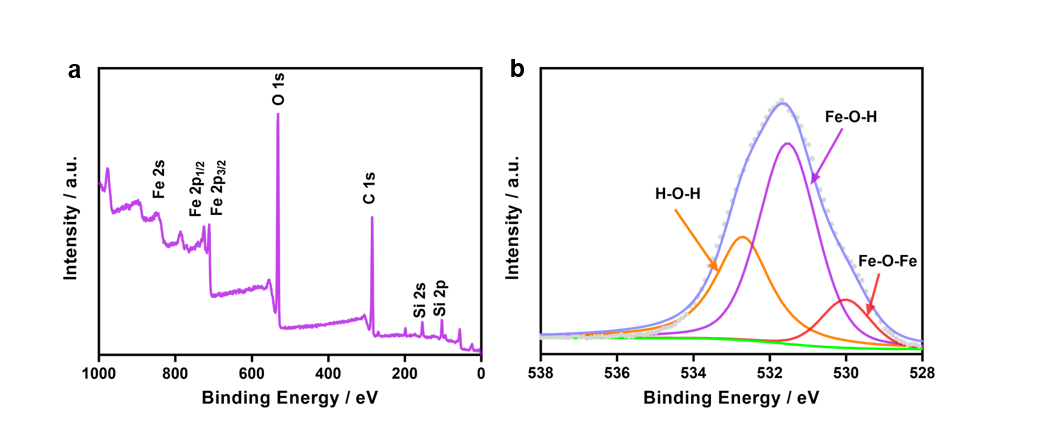


**Supplementary Figure 8. a**) XPS full-survey-scan spectra of MSF and **b**) O 1s core-level spectra of MSF.

**Figure S9.**


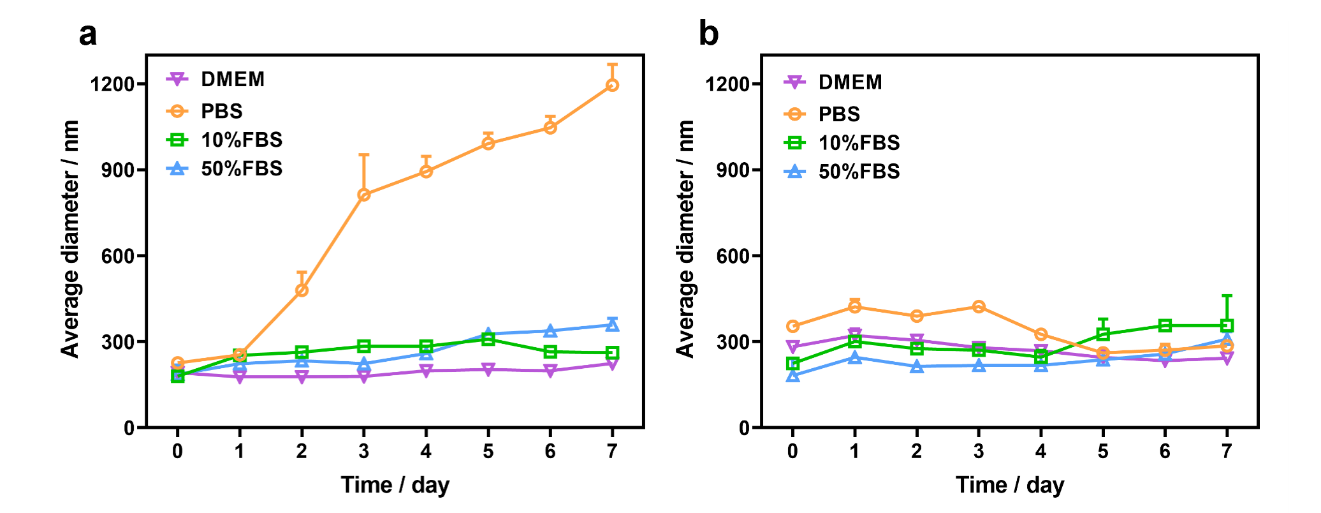


**Supplementary Figure 9.** Changes in the size distribution of **a**) MSF and **b**) MSF@CCM after incubation in different conditions for 7 days.

**Figure S10.**


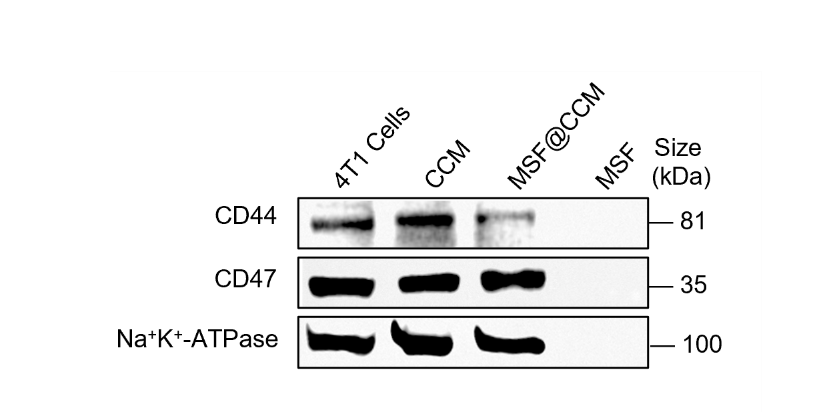


**Supplementary Figure 10.** Membrane proteins of whole 4T1 cells, CCM MSF@CCM and MSF were determined by Western blotting. Na^+^K^+^-ATPase was used as a loading control.

**Figure S11.**

**
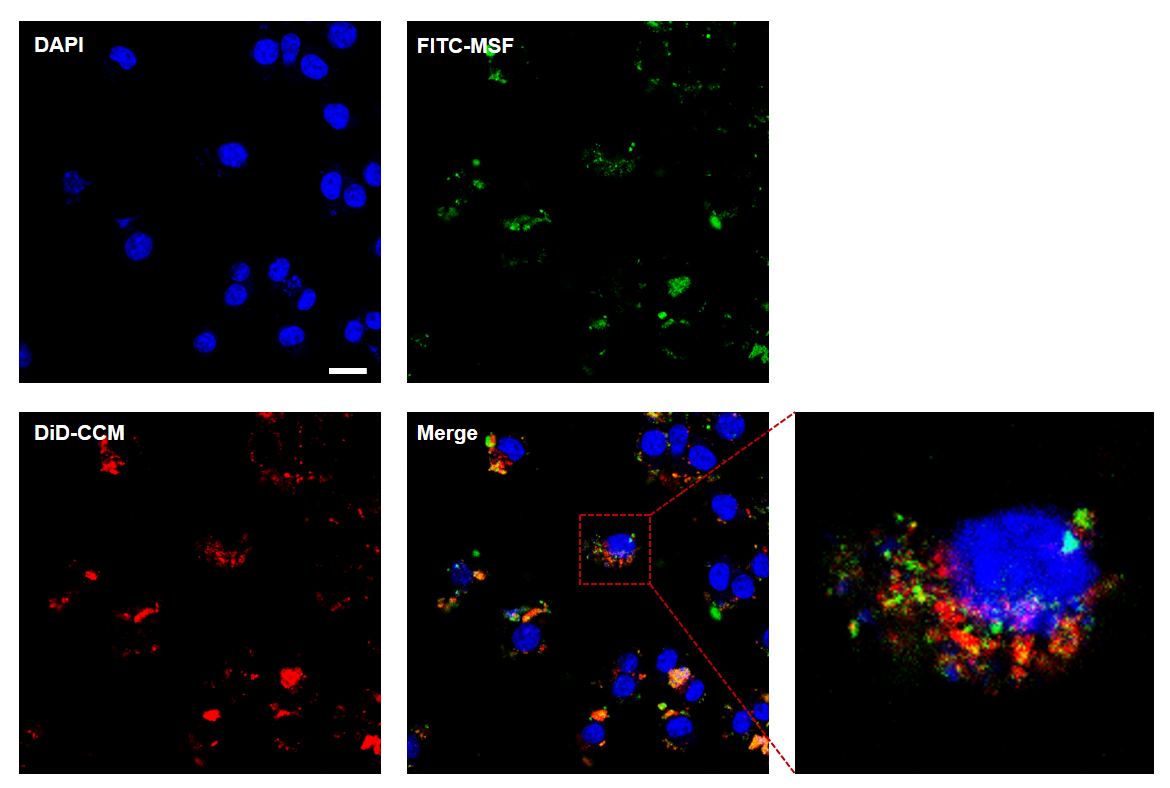
**

**Supplementary Figure 11.** CLSM images showed the co-localization of MSF (labeled with FITC; green) and 4T1 cell membranes (labeled with DiD; red) after being uptaken by 4T1 cells. The MSF@CCM were incubated with 4T1 cells for 4 h. The cell nuclei were stained with DAPI (blue). Scale bar = 20 μm.

**Figure S12.**

**
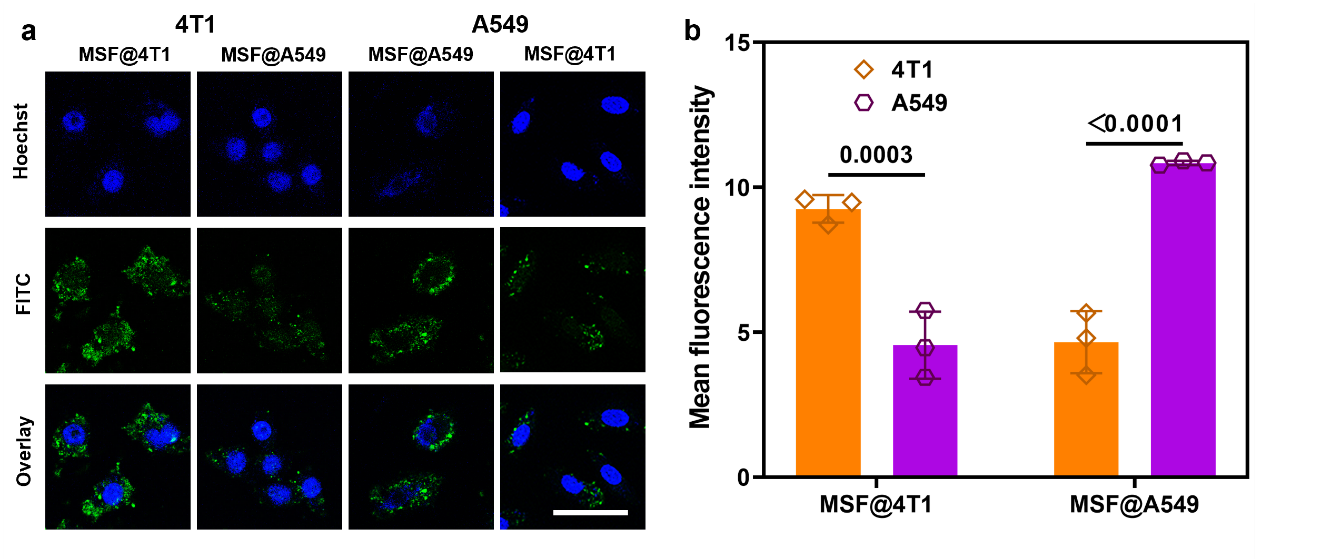
**

**Supplementary Figure 12. a**) CLSM images and **b**) corresponding mean fluorescence intensity of MSF@4T1 (4T1 cell membranes) and MSF@A549 (A549 cell membranes) incubated with 4T1 and A549, cells, respectively. Scale bar = 50 μm. Data are presented as mean ± SD (n = 3). One-way ANOVA with Tukey's multiple comparison was used to calculate statistical differences in **b**.

**Figure S13.**


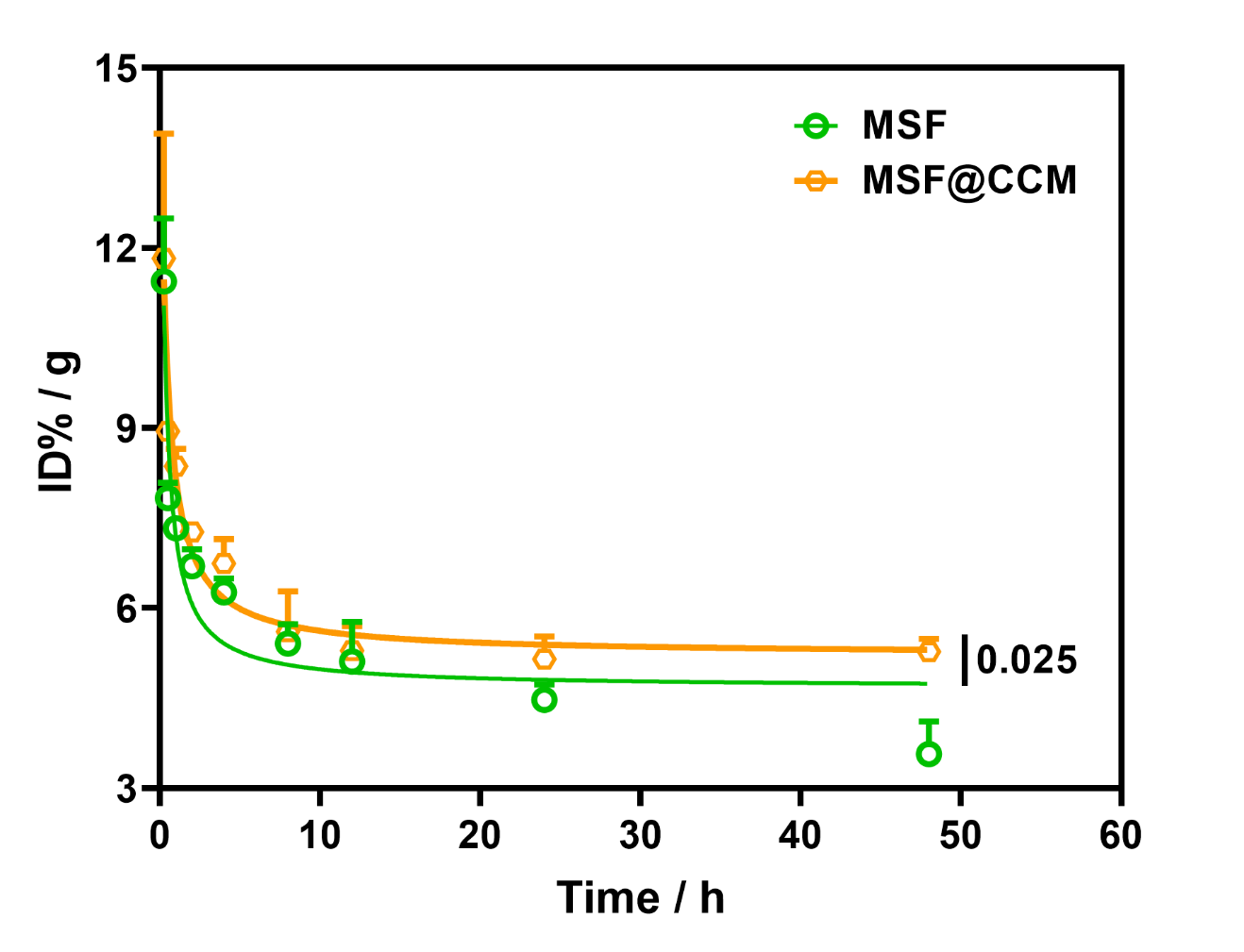


**Supplementary Figure 13.** In vivo pharmacokinetics of MSF and MSF@CCM in tumor-bearing mice. Data are presented as mean ± SD (n = 3). One-way ANOVA with Tukey's multiple comparison was used to calculate statistical differences.

**Figure S14.**

**
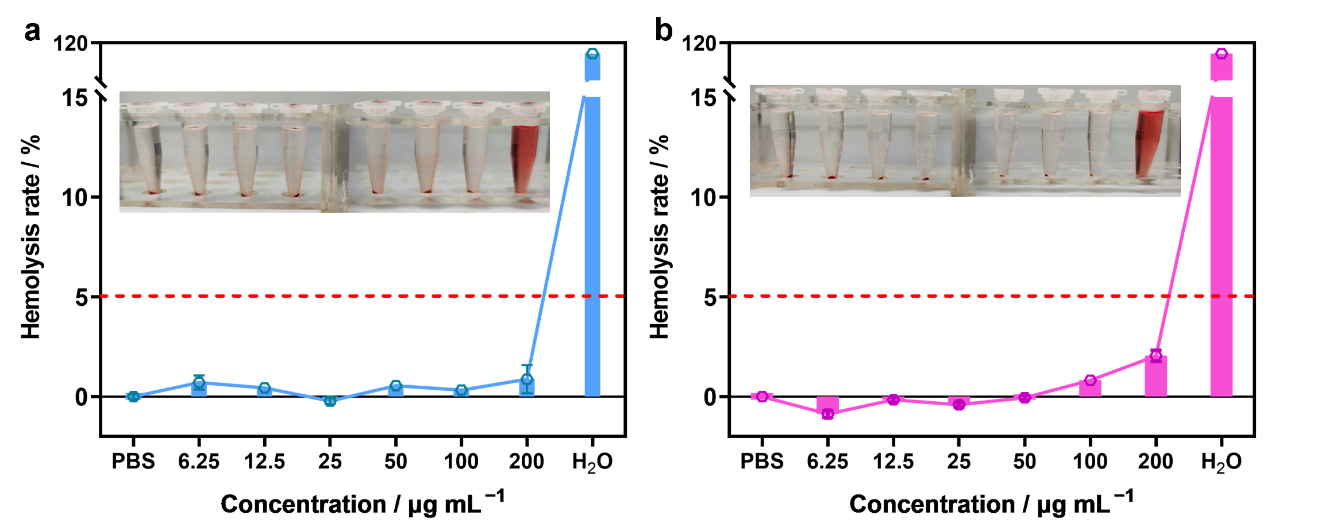
**

**Supplementary Figure 14.** Hemolysis rate of **a**) MSF and **b**) MSF@CCM at different concentrations (6.25, 12.5, 25, 50, 100, 200 μg mL^−1^). Data are presented as mean ± SD (n = 5).

**Figure S15.**

**
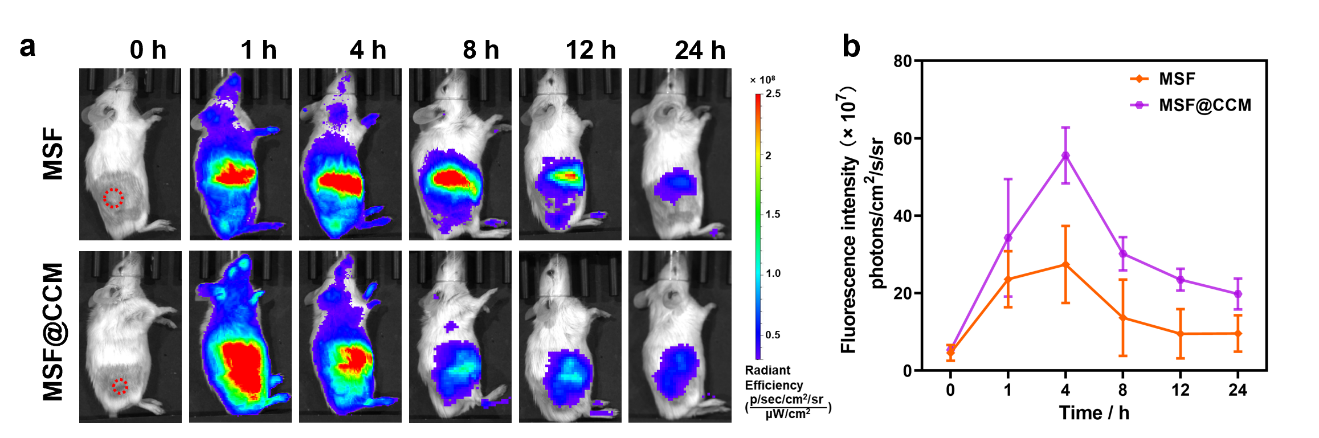
**

**Supplementary Figure 15. a**) Biodistribution of I-MSF and I-MSF@CCM in the mice for 0, 1, 4, 8, 12 and 24 h after *i.v.* injection. Tumors are circled in red. **b**) Corresponding fluorescence intensity of tumors for a). Data are presented as mean ± SD (n = 3).

**Figure S16.**

**
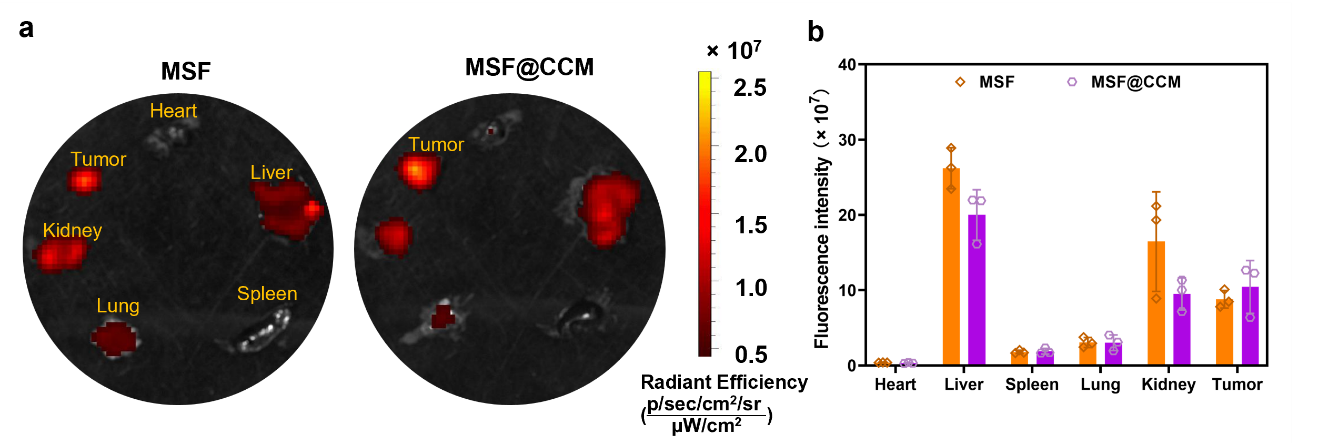
**

**Supplementary Figure 16. a**) Ex vivo fluorescence images and **b**) corresponding fluorescence intensity of major organs and tumors 4 h after *i.v.* injection. Data are presented as mean ± SD (n = 3).

**Figure S17.**

**
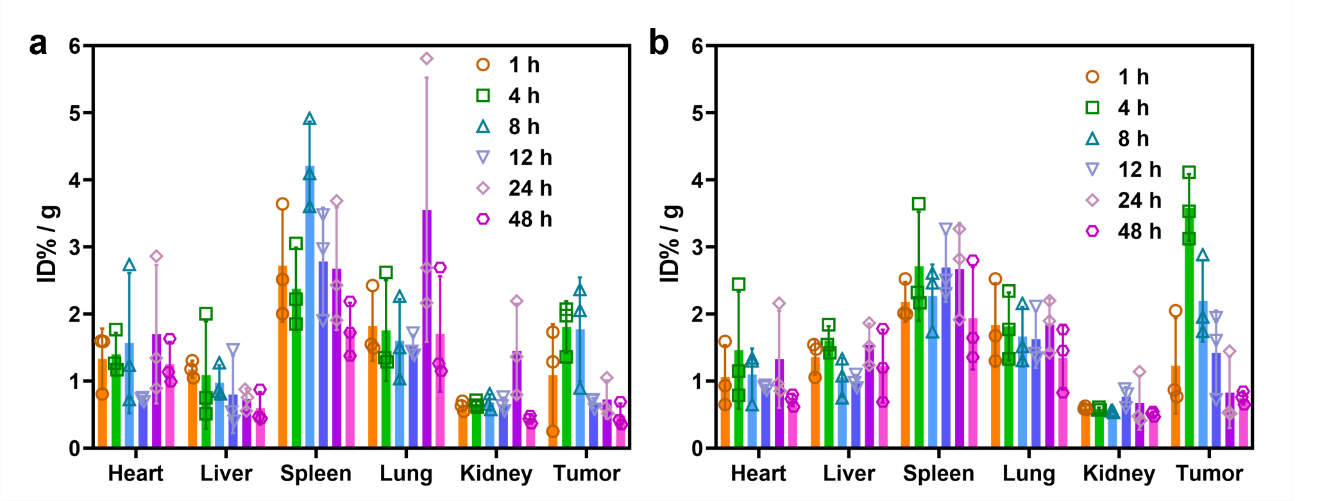
**

**Supplementary Figure 17.** Biodistribution of **a**) MSF and **b**) MSF@CCM in the mice after *i.v.* injection for 1, 4, 8, 12, 24 and 48 h. Data are presented as mean SD (n = 3).

**Figure S18.**


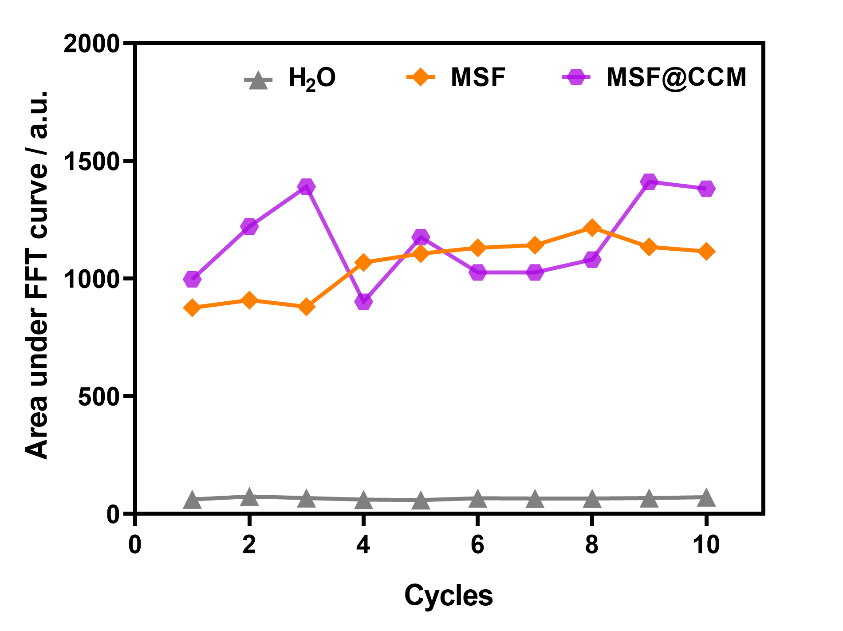


**Supplementary Figure 18.** Integral areas under the FFT curves for different groups over 10 cycles.

**Figure S19.**


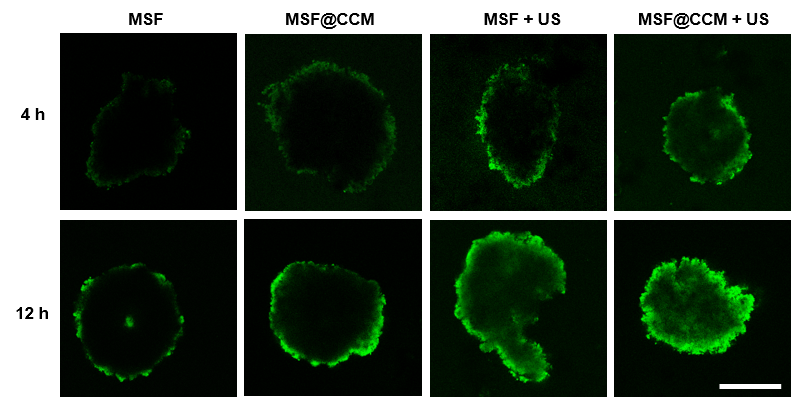


**Supplementary Figure 19.** CLSM images of the tumor penetration of MSF@CCM and MSF in MTSs after different incubation times, with or without US irradiation. Scale bar = 200 μm. US: 1.0 MHz, 1 W cm^−2^, 5 min, 50% duty cycle.

**Figure S20.**


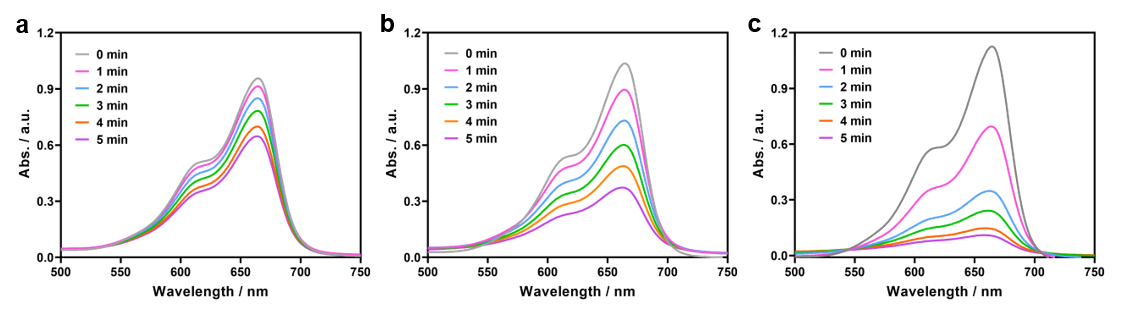


**Supplementary Figure 20.** Time-dependent sono-degradation of MB caused by **a**) MSN, **b**) FeOOH nanodots, and **c**) MSF under US (1.0 MHz, 1.5 W cm^−2^) irradiation.

**Figure S21.**


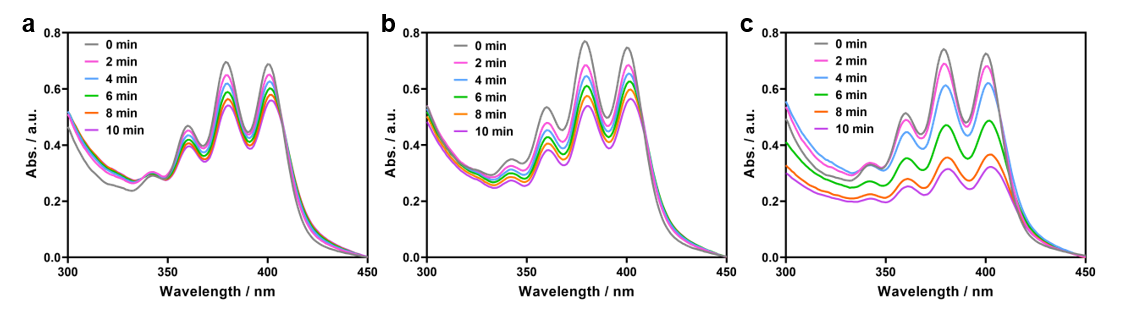


**Supplementary Figure 21.** Time-dependent sono-degradation of DPA caused by **a**) MSN, **b**) FeOOH nanodots, and **c**) MSF under US (1.0 MHz, 1.5 W cm^−2^) irradiation.

**Figure S22.**

**
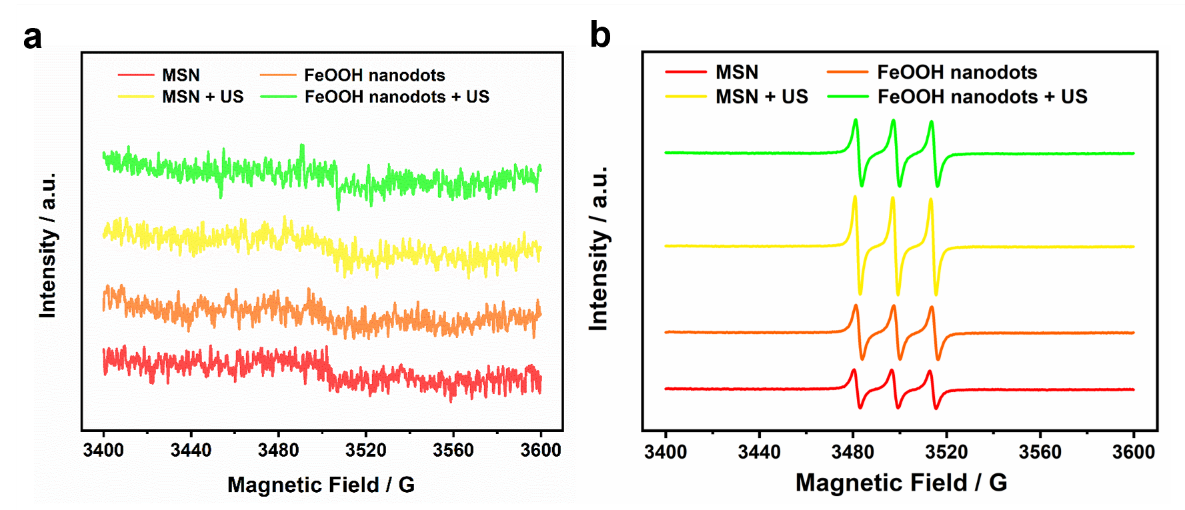
**

**Supplementary Figure 22. a**) ESR spectra of •OH **b**) ^1^O_2_ after different treatments.

**Figure S23.**


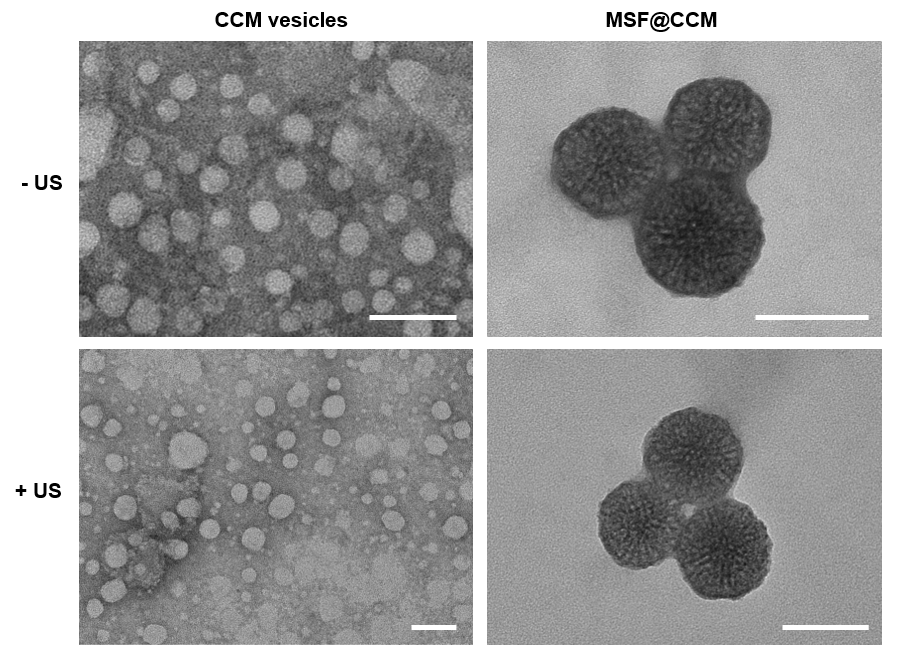


**Supplementary Figure 23.** TEM images of 4T1 CCM-vesicles and MSF@CCM with or without US (1.0 MHz, 1.5 W cm^−2^, 2 min) irradiation. Scale bar = 100 nm.

**Figure S24.**

**
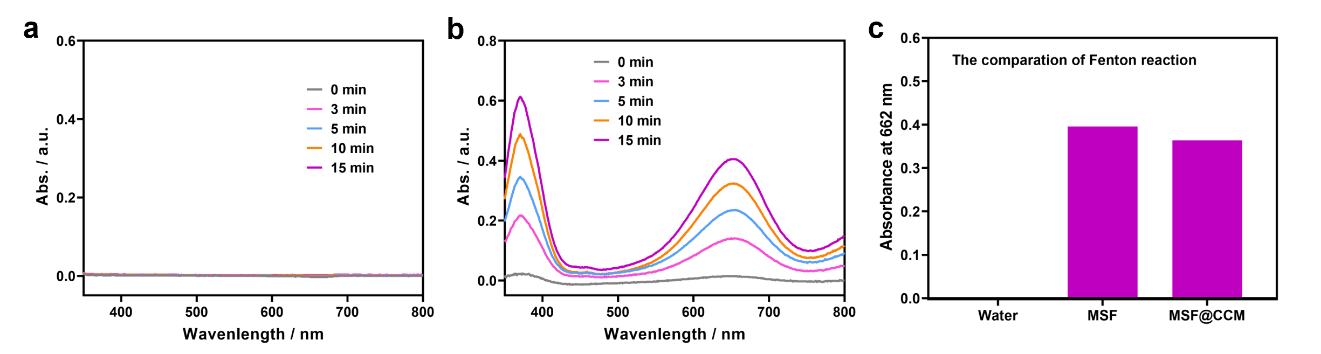
**

**Supplementary Figure 24.** The effect of Fenton reaction of **a**) water and **b**) MSF reflected by the chromogenic reaction of TMB probe. **c**) The comparation of Fenton reaction of water, MSF, and MSF@CCM. The concentration of H_2_O_2_ is 100 µM.

**Figure S25.**


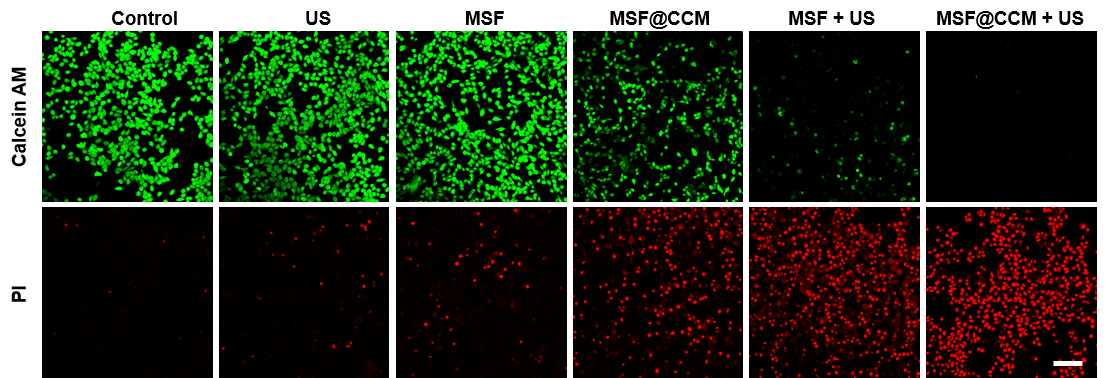


**Supplementary Figure 25.** CLSM images of 4T1 cells co-stained by Calcein AM/PI. Green (Calcein AM), live cells; red (PI), dead cells. MSF and MSF@CCM are 100 μg mL^−1^ respectively; US: 1.0 MHz, 1.5 W cm^−2^, 2 min, 50% duty cycle. Scale bar = 100 μm.

**Figure S26.**

**
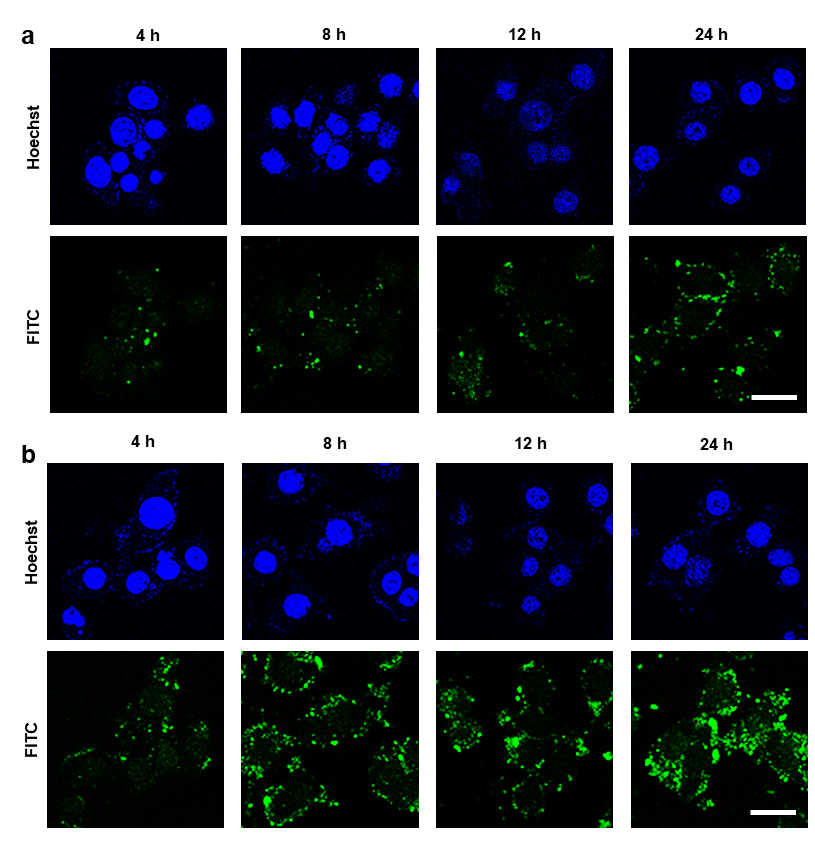
**

**Supplementary Figure 26.** CLSM images of 4T1 cells incubated with **a**) FITC-labeled MSF and **b**) FITC-labeled MSF@CCM for different time points. Scale bar = 25 μm.

**Figure S27.**


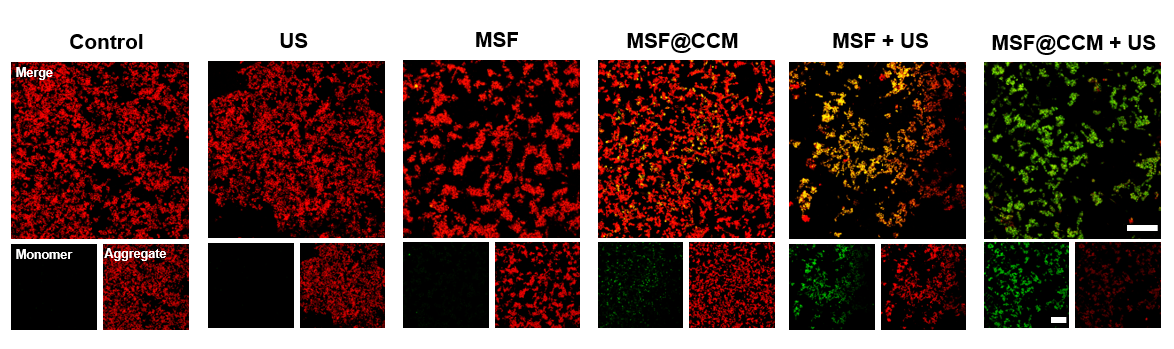


**Supplementary Figure 27.** JC-1 staining was performed on 4T1 cells under different treatment conditions. Scale bar = 200 μm.

**Figure S28.**

**
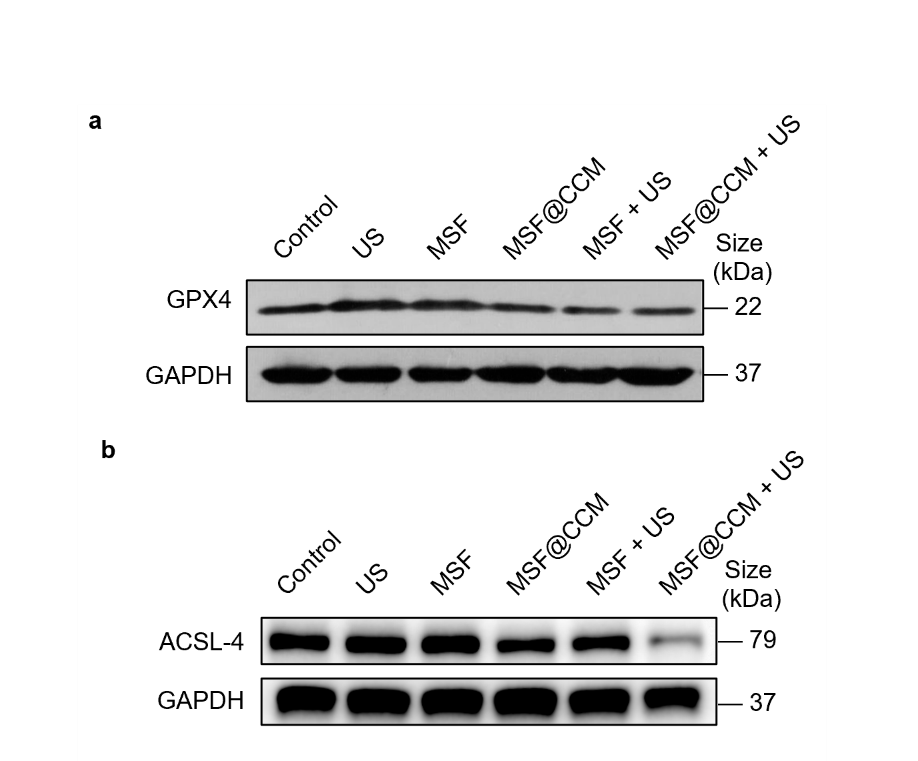
**

**Supplementary Figure 28.** Western blotting analysis the expression of key ferroptosis makers **a**) GPX4 and **b**) ACSL-4 after different treatments. GAPDH was used as a loading control.

**Figure S29.**


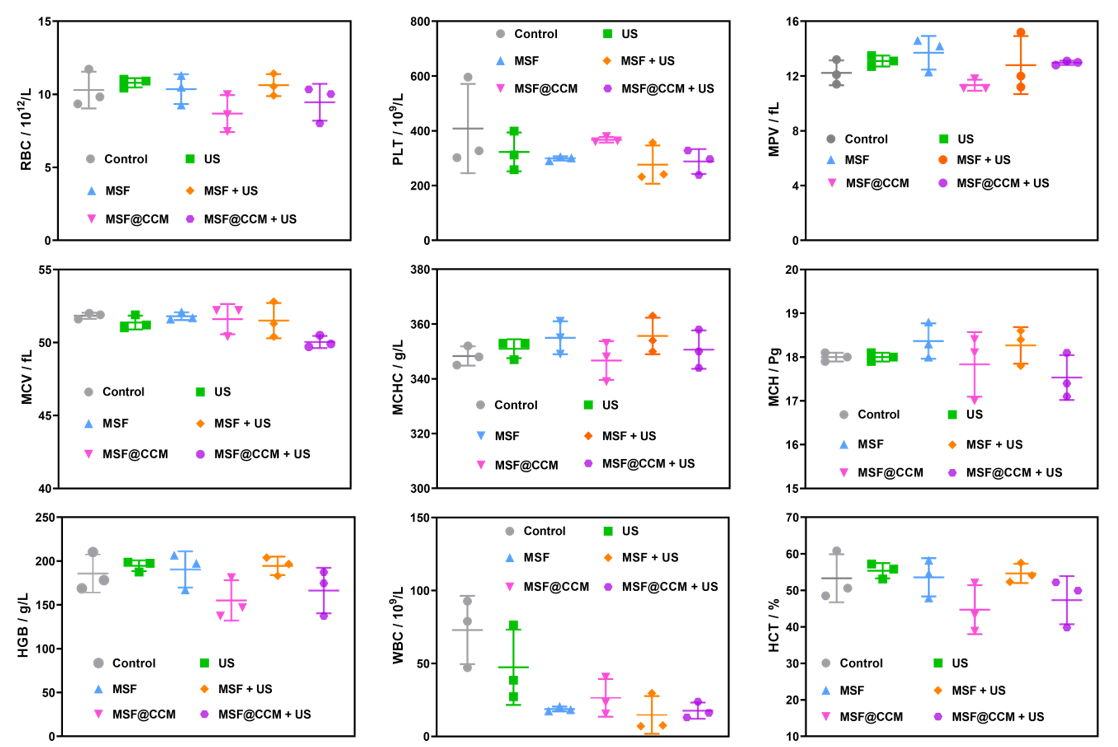


**Supplementary Figure 29.** Complete blood panel analysis of mice collected at 14 days after different treatments. Data are presented as mean ± SD (n = 3).

**Figure S30.**


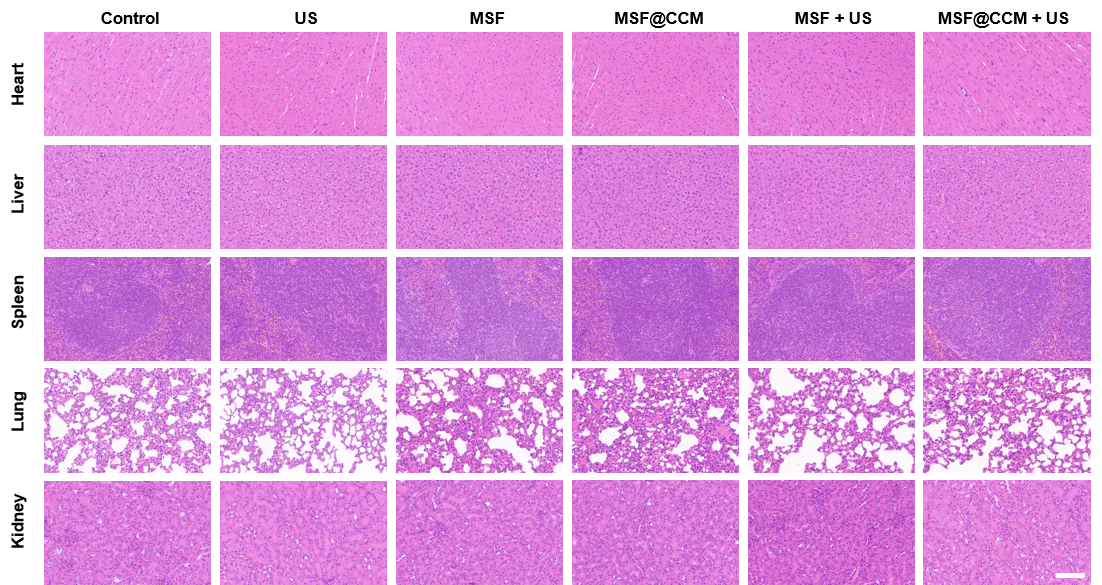


**Supplementary Figure 30.** H&E-stained images of major organs with different treatments. Scale bar = 100 μm.

**Figure S31.**


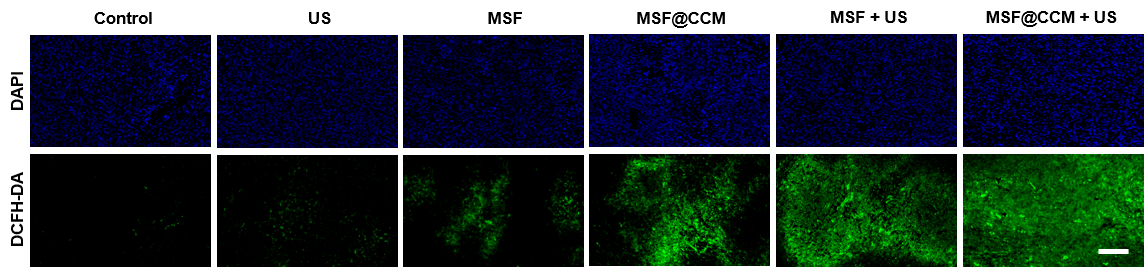


**Supplementary Figure 31.** Fluorescence images of tumor slices collected after treatment for 4 h and stained with DCFH-DA. (green (DCFH-DA), ROS; blue (DAPI), nuclei). Scale bar = 100 μm.

**Figure S32.**


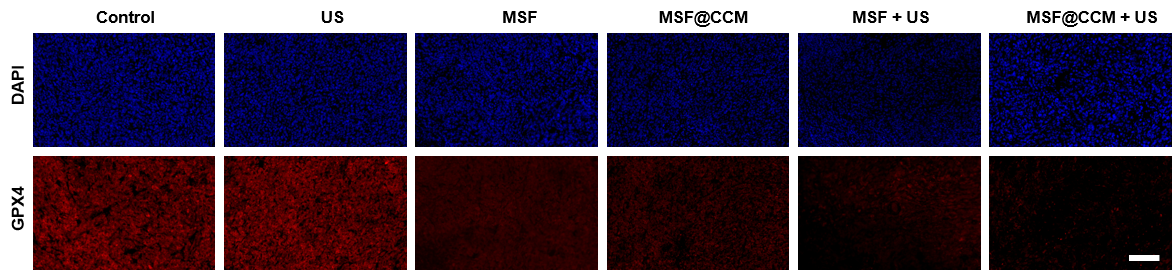


**Supplementary Figure 32.** Immunofluorescence images of GPX4 expression in tumor tissues after different treatments (red: GPX4, blue (DAPI): nuclei). Scale bar = 100 μm.
